# Substituent‐Driven Anion‐Binding Selectivity in Aliphatic Chain‐Substituted 1,2‐Phenylene Urea Macrocycles and Optimized Synthetic Methodology

**DOI:** 10.1002/open.202400469

**Published:** 2025-01-07

**Authors:** Kateřina Svobodová, Václav Eigner, Andrea Brancale, Petra Cuřínová, Michal Himl

**Affiliations:** ^1^ Department of Organic Chemistry University of Chemistry and Technology Prague Technická 5 Prague 6 16628 Czech Republic; ^2^ Department of Solid State Chemistry University of Chemistry and Technology Prague Technická 5 Prague 16628 Czech Republic

**Keywords:** Computational chemistry, Host-guest systems, Macrocycles, Template synthesis, Urea

## Abstract

1,2‐Phenylene tetraurea macrocycles recently attracted attention as self‐assembled channel‐making compounds with high selectivity to chlorides. Here, we report on the introduction of aliphatic chains in the periphery of the 1,2‐phenylene tetraurea macrocycle, which led to deterioration in the ability of the macrocycle to form channels and to a reversal of anion binding preferences in favour of dihydrogen phosphate. In addition, we have developed a new method of synthesis of 1,2‐phenylene tetraurea macrocycle, using a direct click of two diamino ureido derivatives by triphosgene in the presence of chloride template. This approach saves time and eliminates demanding isolation of the non‐cyclic tetrameric intermediates.

## Graphical abstract

Decorating the periphery of the 1,2‐phenylene urea macrocycle with butoxy groups leads to a disruption of the ability of the compound to form self‐assembled channels, which is typical for this class of receptors. For this reason, the molecule adopts a selectivity for dihydrogen phosphate. In addition, a simplified synthetic procedure is presented.

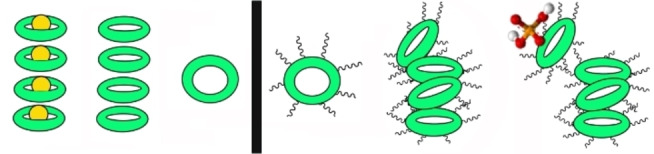

## Introduction

**Figure 1:**
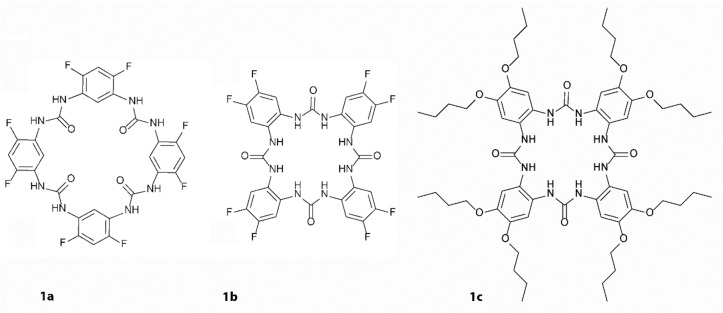
Structures of phenylene‐urea tetramers; **1 a**, **1 b**: previously published compounds, **1 c**: newly designed receptor.

Aromatic ureas represent a class of compounds frequently used in the design of anion receptors in the field of supramolecular chemistry.^[1]^ Urea owes this popularity to its ability to form strong directional hydrogen bonds. Moreover, when the urea based binding sites are suitably oriented in space, further advantages are obtained in the recognition of anion shape.^[2]^ In this respect, cyclic structures have an exclusive position due to their limited entropy, hence providing selectivity based on the size exclusion. Cyclic aromatic ureas were successfully used for recognition of acetate,^[3]^ sulfate,^[4]^ chloride,^[5]^ dicarboxylates,^[6]^ and even for chiral recognition.^[7]^ Long‐term evidence shows that aromatic ureas are much better hydrogen bond donors than aliphatic ones.^[8]^ Hence, the receptor design using direct interconnection of urea motif with phenylenes leads to macrocycles with unique properties. In the case of 1,3‐phenylene tetraurea macrocycles, the oxygen‐in conformation of the cycle confers unexpected cation‐binding affinity to the macrocycles (Figure 1, compound **1 a**).^[9]^ On the other hand, using 1,2‐phenylene for urea group bridging leads to molecules with superior selectivity for chloride anion (Figure 1, compound **1 b**).^[10]^ Such complexes are strong enough to be used for chloride complexation in the aqueous environment^[11]^ or for its transport through the lipid membrane.^[12]^ The crystal structures of these macrocycles are known, explaining their high tendency to aggregate into infinite columns interconnected by hydrogen bonds and π‐π stacking interactions. The complexation of chlorides destabilizes these aggregates and leads to stepwise conformational changes. Chlorides penetrate the channels separating them first into 2 : 1 macrocycle:chloride sandwiches which with increasing chloride load become 1 : 1 complexes. The high loads of chloride result in monomeric 1/2 receptor – chloride complex, with chloride anions on the opposite sides of the macrocycle.^[10]^ Based on this knowledge, we decided for a detailed study of a macrocycle bearing butoxy‐groups which should lead to inherent disruption of potential channels (Figure 1, compound **1 c**) enabling a clearer insight into anion binding principles in these structures.

## Results and Discussion

### Synthesis

**Scheme 1:**
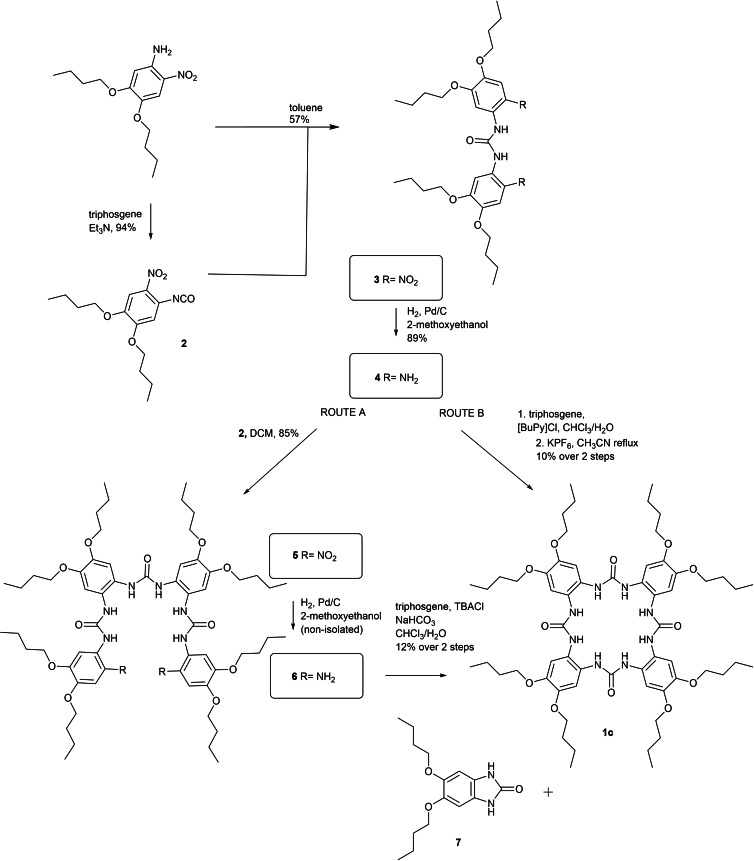
Synthesis of compound **1 c**; route A, already published for **1 b**,^[11]^ and the newly proposed route B.

As a starting compound, 4,5‐dibutoxy‐2‐nitroaniline^[13]^ was used in its toluene solution. Upon addition of triphosgene in the presence of triethylamine, isocyanate **2** was formed in 94 % yield. Ureido derivative **3** was prepared by nucleophilic addition of 4,5‐dibutoxy‐2‐nitroaniline on the isocyanate groups of **2** in 57 % yield. The resulting ureido derivative **3** was reduced catalytically by hydrogen giving diamine **4**. To obtain the target compound, we first used the procedure published for compound **1 b**, route which consists of the gradual building of the macrocycle. In this case, diamine **4** is added as a nucleophile to the isocyanate group of compound **2**, leading to the linear dinitro ureido derivative **5**. The characterization of this compound by NMR methods was complicated by its high flexibility at room temperature. The spectra were thus recorded at 70 °C, where all the movements are faster at NMR time‐scale and the signals are averaged. The Pd catalysed reduction of the nitro‐groups of **5** by hydrogen was somewhat problematic due to very low solubility of this compound in most of the organic solvents. Most of the commonly used solvents (MeOH, EtOH, THF) and their combinations did not give the product at all or in a very small yield. The best results were reached using 2‐methoxyethanol, stirring for 5 days, under 8 Bar of hydrogen. Moreover, acyclic amine **6** was very difficult to purify and characterize. The amphiphilic character of the compound led to high aggregation which compromised the ^1^H NMR spectra, giving very broad signals. The presence of the compound was then confirmed by TLC and indirectly by the subsequent reaction leading to the final product **1 c** after chloride templated cyclization. In accordance with the literature,^[11]^ the chloride anion was used as tetrabutylammonium salt. Due to all of the above drawbacks, a new route (route B, scheme 1) was developed, where the macrocycle is formed directly by Cl^−^ templated cyclization of two diamino ureido derivatives. In this case, ureido derivatives **4** are reacted with triphosgene using chloride anion as a template in the form of butyl pyridinium salt. The BuPy cation has a function in the workup: it can be much more easily removed from the reaction mixture than tetrabutylammonium. Using reflux in acetonitrile in the presence of potassium hexafluorophosphate, the pure macrocycle, without complexed chloride anion was readily obtained. Although the yield of route B was very small, the method itself had some indisputable advantages. Compared to route A, the number of steps was reduced. Compound **1 c** was readily available by precipitation, so the purification of the final molecule becomes easy.

In both routes, an excessive formation of compound **7** was observed. This kind of compound is a common side‐product and formation of similar structures was already published.^[11]^ Compound **7** has an ^1^H NMR spectrum with the same pattern as **1 c** so the structure of **1 c** should be always confirmed by HRMS to prevent confusion with this impurity. The structure of **7** was verified by X‐ray analysis and crystallographic data are available in the SI file (Figure S39).

Unfortunately, we were not able to obtain crystals of **1 c**. Clearly, the addition of aliphatic chains into the macrocycle led to a flexible structure. In this context, the molecule also gained better solubility in lipophilic solvents; in contrast to **1 b**,**1 c** can be dissolved even in chloroform.

### Anion Complexation

**Figure 2:**
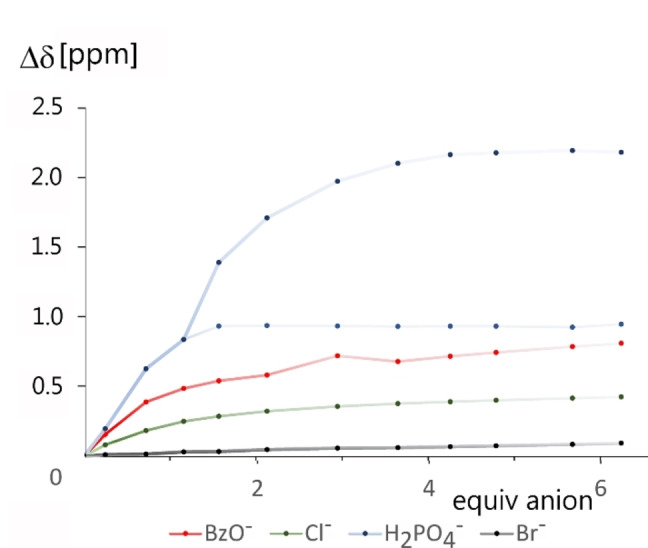
Titration curves of **1 c** with anions, ^1^H NMR, 25 °C, 400 MHz, DMSO‐*d_6_*.

According to ^1^H NMR measurements, in both CDCl_3_ and DMSO‐*d_6_* solutions, **1 c** forms aggregates.^[14]^ These aggregates cause line broadening of ^1^H NMR signals at ambient temperature. Heating of **1 c** in its DMSO‐*d_6_* solution to 70 °C leads to faster moving of the molecules and signal sharpening to the point that the *J*‐ splitting becomes visible (Figure S25, S26). After these preliminary measurements we monitored the behaviour of **1 c** in the presence of inorganic anions; Cl^−^, Br^−^, BzO^−^, H_2_PO_4_^−^, NO_3_^−^ (Figure 2 and S30–S33). These anions were added into 2.7 mM DMSO‐*d_6_* solution of **1 c** as tetrabutylammonium salts under the conditions of ^1^H NMR titration.^[15]^ As the anion complexation is complicated by receptor aggregation, the titration curves reveal multiple ongoing processes (Figure 2). The aggregates seem to be quite stable and, before anion complexation, the network of hydrogen bonds connecting individual macrocycles in the aggregates must be altered. At the higher anion/receptor ratios, the 1 : 2 stoichiometry complexes are formed. Considering all the above features, the reliable determination of association constants by NMR becomes difficult. On the other hand, titrations in UV‐Vis can be performed at a concentration two order of magnitude lower than ^1^H NMR, where the problem of macrocycle self‐aggregation is negligible. As supported by a dilution experiment, compound **1 c** at the concentrations below 5*10^−4^ M behaves linear on dilution (Figure S34). Therefore, the UV‐Vis titrations were performed at 0.04 mM DMSO solutions of **1 c**, adding the corresponding aliquots of DMSO solutions of anions. The complexation constants were calculated using program Bindfit^[16]^ and the experimental data was fitted as 1 : 2 non‐cooperative complexes (Figure S35–S38). This choice was based on the fact, that the experimental data for all the tested anions best matched this fit. The results are provided in Table 1 as overall binding constants ß_1:2_.

**Table 1:** Table Complexation constants of **1 c** towards TBA^+^ salts of various anions (UV‐Vis, 25 °C, DMSO).

Anion	NO_3_^−^	Br^−^	Cl^−^	BzO^−^	H_2_PO_4_^−^
ß_1:2_*10^3^ [M^−2^] error [%]	0 0	0.22 0.5	1.67 11	17.18 2.5	57.97 6

The above measurements describe well the complexation behavior of **1 c**. In the case of nitrate, no changes in the ^1^H NMR or UV‐Vis spectra of **1 c** were observed, no complexation occurred. The halides made with **1 c** 1 : 2 complexes and showed preference of chloride over bromide. Compared to published **1 b**/Cl^−^ complexes,^[11]^ in the case of **1 c** no sandwich complexes with 2 : 1 macrocycle‐anion stoichiometry on the way to 1 : 1 complex were observed.

**Figure 3:**
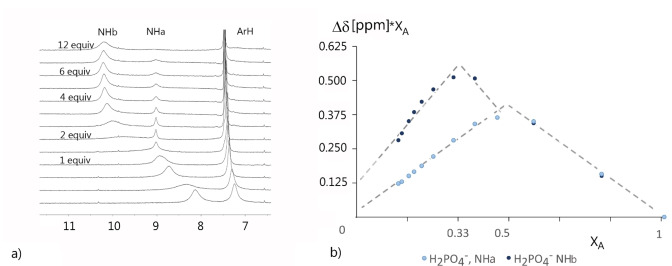
^1^H NMR shifts of NH groups of 1c upon titration with TBA dihydrogenphosphate and their plot against molar fractions, ^1^H NMR, 25 °C, 400 MHz, DMSO‐*d_6_*.

With benzoate, even stronger complexes were formed. Nevertheless, the most interesting behavior was observed in ^1^H NMR during the dihydrogen phosphate complexation (Figures 2 and 3). After addition of one equivalent of anion, the signal of NH groups split into two. One of them (NH_a_) remained at the position corresponding to the strong 1 : 1 complex. The newly formed NH signal (NH_b_) moved with further addition of dihydrogen phosphate to the lower field, suggesting the formation of the 1 : 2 complex.

**Figure 4:**
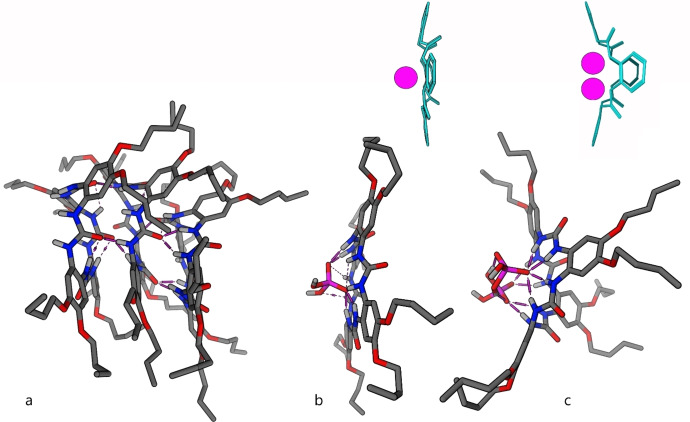
Molecular dynamics calculations. a) Free **1 c** in DMSO. b) 1 : 1 complex of **1 c** with dihydrogen phosphate. c) 1 : 2 complex of **1 c** with dihydrogen phosphate.

The experimental data were compared to the data obtained from a series of molecular dynamic simulations. The molecular dynamics data obtained with the free receptor **1 c** in DMSO (Figure 4a) demonstrate the above described formation of aggregates. The butoxy chains bring disorder into the aggregate pattern and significantly distort the stacked macrocycles from planarity. This extra disorder added to the macrocycle periphery can justify the compromised molecular stacking into channels, and thus the difficult compound crystallization, and the ^1^H NMR spectra with chemical exchange burden at the room temperature.

Compared to **1 b**, the **1 c** macrocycle binding site is sterically hindered by the butoxy groups, which prevents the penetration of anions into aggregates and the sandwich complexes formation. Therefore, upon formation of the complex, the aggregate must be disrupted and the macrocycle released. This necessarily leads to energy demanding hydrogen bonds reorganization, which lowers the efficiency of **1 c**, but increases its selectivity. Among the tested anions, dihydrogen phosphate is shape‐favored. Its 1 : 1 complex with **1 c** can be very stable, as all the NHs of the macrocycle can be involved in anion binding (Figure 4b). In this case, the macrocycle is rather flat and symmetrical, with all the NH groups having one broad signal. The addition of more equivalents of dihydrogen phosphate causes formation of the 1 : 2 complex. In the timescale of molecular dynamics calculation, no complexes with anions on the opposite sides of the macrocycle were observed. However, it is possible to observe two phosphates switching their places on the same side of the macrocycle, remaining in close proximity of the complexation site (Figure 4c). At the same time, the macrocycle conformation bends, with two aromatic cores in the plane of the original macrocycle and two cores pinched, facing each other. This is in an agreement with the experimental data, where sequential increasing of dihydrogen phosphate leads to the separation of the NH signals and extensive formation of the 1 : 2 complex with different conformation. It should be noted that the experimental data indicate a high stability of the 1 : 1 complex, because even at high loading with dihydrogen phosphate the signal belonging to this complex is still evident. The 1 : 1 complex is also significantly present in the molecular dynamics simulations that present an excess of dihydrogen phosphate in the system.

## Conclusions

The 1,2‐phenylene tetraurea macrocycle decorated at the outer rim with butoxy groups was synthesized and its anion binding properties were thoroughly tested. The introduction of butoxy groups into the structure led to the enhanced selectivity of this receptor to the dihydrogen phosphate anion. The data obtained from molecular dynamic simulation indicate that the influence of butoxy groups on the anion binding properties of the macrocycle are not based on the electronic effects of these groups, but more on their steric demands. The steric burden they bring into the macrocycle prevents efficient macrocycle folding into channels and also the formation of sandwich complexes, otherwise so common for this type of macrocycles. Without folding, the selectivity principle is mainly based on shape recognition, where dihydrogen phosphate is favored due to possible bonding to all the available NH groups. By changing the conformation of the macrocycle, the binding of two phosphate molecules on the same side of the macrocycle is possible. In addition, by optimizing the synthesis, a more efficient method for obtaining tetraurea‐based macrocycles was developed, which significantly shortens and simplifies the synthesis compared to previously published methods.

## Essential Experimental Procedures/Data

### Materials and Methods

Reagents were purchased from commercial sources and used without further purification. Anhydrous solvents were dried by standard procedures; DCM was dried over calcium hydride. For the complexation studies, anions were used in the form of their tetrabutylammonium salts The ^1^H (400 MHz), ^13^C (101 MHz) spectra were recorded using a Bruker Avance 400 spectrometer at 25 °C. Used solvents (DMSO‐*d_6_*, chloroform‐d) were stored over molecular sieves. The ^1^H and ^13^C NMR spectra were referenced to the line of the solvent (δ/ppm; δH/δC: DMSO‐*d_6_* 2.50/39.52, chloroform‐*d*, 7.26/77.16). To assign all proton and carbon signals, a combination of 1D and 2D experiments (H,C‐HSQC and H,C‐HMBC) was used. The high‐resolution mass spectra (HRMS) were measured on a MicrOtof III spectrometer (Bruker) with electrospray (ESI) ionisation source in positive mode. For calibration of accurate masses, ESI‐APCI Low Concentration Tuning Mix (Agilent) was used. The isotope profiles were calculated using freely available software EnviPat Web2.4.^[17]^ Melting points were measured on a hot stage microscope Kofler (KB T300) and were not corrected. UV‐Vis spectra were measured on the Cintra 20 spectrometer (GBC Scientific Ltd., Australia). All UV‐Vis spectra were recorded in the wavelength region from 285 to 600 nm, with 0.5‐nm steps in 10‐mm cuvettes. All molecular modelling experiments were performed on a custom‐made machine with Intel i9‐12900 K x 24, NVIDIA RTX A5000, running Ubuntu 22.04. Molecular Operating Environment (MOE) 2019.10S9 and the Schrödinger suite (release 2021‐2) S10 were used as the main molecular modelling software packages.^[18]^ The single receptors were built in MOE, and energy was minimised using the OPLS4 force field in Maestro. Molecular dynamics simulations were performed using Desmond. The simulation box had an 87 Å cubic shape, and a density of 1.5 g/cm^3^. All simulations were run for 1000 ns, with a 2 fs time step, in the NPT ensemble, with constant temperature (300 K) and pressure (1 atm). All other parameters were set using the Desmond default values. Images were created using PyMOL. Characterization data of the prepared compounds are provided in the attached Supporting Information file (Figure S1–S29).

### Synthesis

#### 4,5‐Dibutoxy‐2‐nitrophenylisocyanate 2

A solution of triphosgene (549 mg, 1.95 mmol) in anhydrous toluene (50 mL) was placed into a 250 mL round bottom flask. To the stirred mixture, a solution of 4,5‐dibutoxy‐2‐nitroaniline (576 mg, 1.94 mmol) in dry toluene (50 mL) was added dropwise, followed by a dropwise addition of solution of triethylamine (0.57 mL, 413 mg, 4 mmol) in anhydrous toluene (20 mL). The reaction mixture was stirred overnight (RT, inert atmosphere). Triethylammonium hydrochloride was filtered off and toluene was evaporated under reduced pressure giving compound **2** as an orange‐yellow solid (564 mg, 94 %).

^1^H NMR (CHCl_3_, 400 MHz): 7.67 (s, 1H, ArH), 6.58 (s, 1H, ArH), 4.08–4.00 (m, 4H, 2xO‐CH_2_), 1.86–1.80 (m, 4H, 2xO‐CH_2_C*H*_2_−), 1.54–1.46 (m, 4H, 2x −C*H*_2_CH_3_, 1.02–0.95 (m, 6H, 2x CH_3_) ppm. ^13^C NMR (CHCl_3_, 101 MHz): 155.3, 146.9, 134.4, 126.8, 124.4, 110.3, 109.7, 69.9, 31.4, 19.6, 14.3 ppm.

Mp 93.5–94.5 °C

HRMS (APCI+) calc for [C_15_H_20_N_2_O_5_+H]^+^: 309.1445, found 309.1438, [M+H]^+^

#### Compound 3

4,5‐dibutoxy‐2‐nitrophenylisocyanate (536 mg, 1.74 mmol) was dissolved in 100 mL of anhydrous toluene. 4,5‐dibutoxy‐2‐nitroaniline (432 mg, 1.53 mmol) was added to the stirred solution. The mixture was refluxed for 3 days and then partially evaporated under reduced pressure to a saturated solution. The product was precipitated from the mixture by MeOH and collected by filtration under reduced pressure giving 3 as a yellow solid (512 mg, 57 %).

^1^H NMR (CDCl_3_, 400 MHz): 10.53, (s, 2H, NH), 8.11 (s, 2H, ArH), 7.67 (s, 2H, ArH), 4.15 (t, 4H, *J=6.45 Hz*, O‐CH_2_), 4.02 (t, 4H, J=6.45 Hz, O‐CH_2_), 1.88–1.78 (m, 8H, 4x O‐CH_2_C*H*_2_−), 1.56–1.45 (m, 8H, 4x C*H*_2_CH_3_, 1.03–0.96 (m, 12H, 4x CH_3_) ppm.^13^C NMR (CDCl_3_, 101 MHz): 156.3, 151.5, 144.1, 132.6, 128.3, 108.9, 103.4, 69.4, 31.1, 19.3, 13.9 ppm.

Mp 142.8–145.5 °C

HRMS (ESI+) calc for [C_29_H_42_N_4_O_9_+H]^+^: 591.3024, found 591.3018 [M+H]^+^

#### Compound 4

Compound **3** (500 mg, 0.85 mmol) was dissolved in 2‐methoxyethanol (50 mL) and hydrogenated in the presence of Pd/C (10 %, 61 mg) in an autoclave at 8 Bar of hydrogen. The mixture was stirred at RT overnight. The catalyst was filtered off and rinsed with boiling CHCl_3_ (3x30 mL). The filtrate was evaporated under reduced pressure to give the final product as a grey‐brown solid (401 mg, 89 %).

^1^H NMR (DMSO‐*d_6_* 400 MHz): 7.52 (s, 2H, NH), 6.90 (s, 2H, ArH), 6.39 (s, 2H, ArH), 4.45 (bs, 4H, 2xNH_2_), 3.83 (t, 4H, *J=6.50 Hz*, O‐CH_2_), 3.77 (t, 4H, *J=6.51 Hz*, O‐CH_2_), 1.64–1.57 (m, 8H, 4x O‐CH_2_C*H*_2_−), 1.45–1.32 (m, 8H, 4x C*H*_2_CH_3_, 0.95–0.82 (m, 12H, 4x CH_3_) ppm. ^13^C NMR (DMSO‐*d_6_*, 101 MHz): 154.2, 146.4, 140.1, 134.7, 118.0, 113.5, 103.6, 69.6, 68.2, 31.0, 15.8, 13.7 ppm.

Mp 164.7–167.7 °C

HRMS (ESI+) calc for [C_29_H_46_N_4_O_5_+H]^+^: 531.3541 found 531.3537 [M+H]^+^

#### Compound 5

To the solution of compound **4** (181 mg, 0.34 mmol) in anhydrous dichoromethane (45 mL), compound **2** was added (232 mg, 0.75 mmol) and the reaction mixture was stirred at the ambient temperature for 4 days. The solvent was then removed under reduced pressure. The crude reaction mixture was chromatographed (ethyl acetate/chloroform 1/20) to give compound **5** (496 mg, 85 %) as a yellow solid. As the compound revealed very mobile structure, the NMR spectra were recorded at 70 °C.

^1^H NMR (400 MHz, DMSO‐*d_6_*, 70 °C): 9.89 (s, 2H, NH), 8.93 (s, 2H, Ar−H), 8.09 (m, 2H, NH), 8.06 (s, 2H, NH), 7.54 (s, 2H, Ar−H), 7.33 (s, 2H, ArH), 7.04 (s, 2H, ArH), 4.02–3.96 (m, 8H, 4xO‐CH_2_), 3.93 (t, *J=6.9 Hz*, 4H, 2xO‐CH_2_), 3.91 93 (t, *J=6.9 Hz*, 4H, 2xO‐CH_2_), 1.73–1.65 (m, 16H, 8xO‐CH_2_C*H*_2_), 1.48–1.41 (m, 16H, 8xC*H*_2_CH_3_), 0.96–0.91 (m, 24H, 8xCH_3_). ^13^C NMR (101 MHz, DMSO‐*d_6_*, 70 °C): 154.8 (C−O−), 153.6 (C=O), 152.8 (C=O), 146.2, 144.8, 142.5 (C−O−), 132.5 ((C‐NO2), 128.1, 126.5, 122.3 (C−NH), 112.6, 110.6, 109.1, 104.3 (4xC−H), 68.8, 68.7, 68.6, 68.3 (O−CH_2_), 30.69, 30.68, 30.4, 30.1 (O‐CH_2_ *C*H_2_), 18.37, 18.36, 18.30, 18.2 (*C*H_2_CH_3_), 13.22, 13.21, 13.19, 13.11 (CH_3_).

Mp 194–196.5 °C

HRMS (ESI+) calc for [C_59_H_86_N_8_O_15_+Na]^+^: 1169.6104 found 1169.6103 [M+Na]^+^

#### Compound 6

Compound **5** (211 mg, 0.18 mmol) was dissolved in 2‐methoxyethanol (30 mL) and hydrogenated in the presence of Pd/C (10 %, 106 mg) in an autoclave at 8 Bar of hydrogen. The mixture was stirred at RT for 5 days. The catalyst was filtered off over silica giving **6** as a brownish syrup. As the attempts on isolation of the pure product **6** failed, the crude product was used immediately for the next step.

#### Compound 7

Compound **7** is an unwanted by‐product in the formation of **1 c**.

^1^H NMR (DMSO‐*d_6_*, 400 MHz, 70 °C): 10.20 (s, 2H, NH), 6.72 (s, 2H, ArH), 3.88 (t, 4H, *J=6.45 Hz*, O−CH_2_), 1.75–1.68 (m, 4H, 2x O‐CH_2_C*H*_2_−), 1.48–1.38 (m, 4H, 2x C*H*_2_CH_3_, 0.93 (t, 6H, *J=6.95 Hz*, 2x CH_3_) ppm. ^13^C NMR (DMSO‐*d_6_*, 101 MHz): 157.9, 145.6, 122.6, 98.6, 70.3, 31.6, 19.3, 13.9 ppm.

HRMS (ESI+) calc for C_15_H_22_N_2_O_3_+Na]^+^: 301.1523 found 301.1518 [M+Na]^+^

#### Compound 1 c

##### Route A

Compound **6** (100 mg, 0.09 mmol) and tetrabutylammonium chloride (182 mg, 0.65 mmol) were stirred for 5 min in 15 mL of chloroform. In a separate vial, a solution of NaHCO_3_ (33 mg, 0.39 mmol) in 15 mL of distilled water was prepared. This solution was added to the solution of **6** followed by the addition of triphosgene (11 mg, 0.039 mmol). The mixture was stirred for 3 days at the ambient temperature. The layers were separated and the organic layer was evaporated to dryness. The crude mixture was purified by flash chromatography on a reversed phase (C18 column, gradient elution from 20 to 100 % of acetonitrile in water). The product was obtained as a brownish solid (12 mg, 12 %).

##### Route B

Compound **4** (810 mg, 1.52 mmol) was dissolved in 120 mL of chloroform together with [BuPy]Cl (910 mg, 5.3 mmol) and the mixture was stirred for 5 min. The solution of NaHCO_3_ (520 mg, 6.19 mmol) in 120 mL of distilled water was added followed by the addition of trifosgene (173 mg, 0.59 mmol). The mixture was stirred for 3 days at the ambient temperature and the layers were separated. The organic layer was evaporated and the residue was dissolved in 150 mL of acetonitrile. KPF_6_ (2 mg, 10.6 mmol) was added and the solution was refluxed for 2 hours. Acetonitrile was evaporated, the residue was dissolved in chloroform (150 mL) and extracted with water (3x 200 mL). The organic phase was dried by magnesium sulphate and after partial removal of the solvent the pure product was precipitated with hexane (brownish solid 85 mg, 10 %). As the compound revealed very mobile structure, the NMR spectra were recorded at 75 °C.

^1^H NMR (400 MHz, DMSO‐*d_6_* 75 °C): 8.24 (s, 8H, NH), 7.33 (s, 8H, Ar−H), 3.96–3.93 (m, 16H, 8xO‐C*H_2_*), 1.73–1.68 (m, 16H, 8xO‐CH_2_C*H*_2_), 1.50–1.45 (m, 16H, 8xC*H*_2_CH_3_), 0.96 (t, *J=7.4 Hz*, 24H, 8xCH_3_). ^13^C NMR (101 MHz, DMSO‐*d_6_*, 75 °C): 153.5 (C=O), 144.7 (C−O−), 124.2 (C−NH), 111.2 (C−H), 68.9 (O−*C*H_2_), 30.8 (O−CH_2_ *C*H_2_), 18.3 (*C*H_2_CH_3_), 13.4 (CH_3_),

Mp 218.5–220.0 °C

HRMS (ESI+) calc for C_60_H_88_N_8_O_12_+Na]^+^: 1135.6414 found 1135.6413 [M+Na]^+^

## Conflict of Interests

The authors declare no conflict of interest.

## Supporting information

As a service to our authors and readers, this journal provides supporting information supplied by the authors. Such materials are peer reviewed and may be re‐organized for online delivery, but are not copy‐edited or typeset. Technical support issues arising from supporting information (other than missing files) should be addressed to the authors.

Supporting Information

## Data Availability

The data that support the findings of this study are available in the supplementary material of this article.
