# Supplementary material for: Substituent‐Driven Anion‐Binding Selectivity in Aliphatic Chain‐Substituted 1,2‐Phenylene Urea Macrocycles and Optimized Synthetic Methodology
Source: ChemistryOpen. 2025 Jan 7;14(8):e202400469. doi: 10.1002/open.202400469 (PMC12957884; doi:10.1002/open.202400469)
Supplement: Supplementary file 1 — Supporting Information [file OPEN-14-e202400469-s001.pdf]

ChemistryOpen

Supporting Information

Substituent-Driven Anion-Binding Selectivity in Aliphatic Chain-Substituted 1,2-Phenylene Urea Macrocycles and Optimized Synthetic Methodology

Kateřina Svobodová, Václav Eigner, Andrea Brancale, Petra Cuřínová, and Michal Himl*

Supporting information for

Substituent-Driven Anion-Binding Selectivity in Aliphatic Chain-Substituted 1,2-Phenylene Urea Macrocycles and Optimized Synthetic Methodology

Kateřina Svobodová,[a] Václav Eigner,[b] Andrea Brancale,[a] Petra Cuřínová,[a] and Michal Himl*[a]

[a] Msc. K. Svobodová, Dr. P. Cuřínová, Prof. A. Brancale, Dr. M. Himl
Department of Organic Chemistry
University of Chemistry and Technology Prague
Technická 5, Prague 6, 16628, Czech Republic
[b] Dr V. Eigner
Department of Solid State Chemistry
University of Chemistry and Technology Prague
Technická 5, Prague 6, 16628, Czech Republic
E-mail: michal.himl@vscht.cz

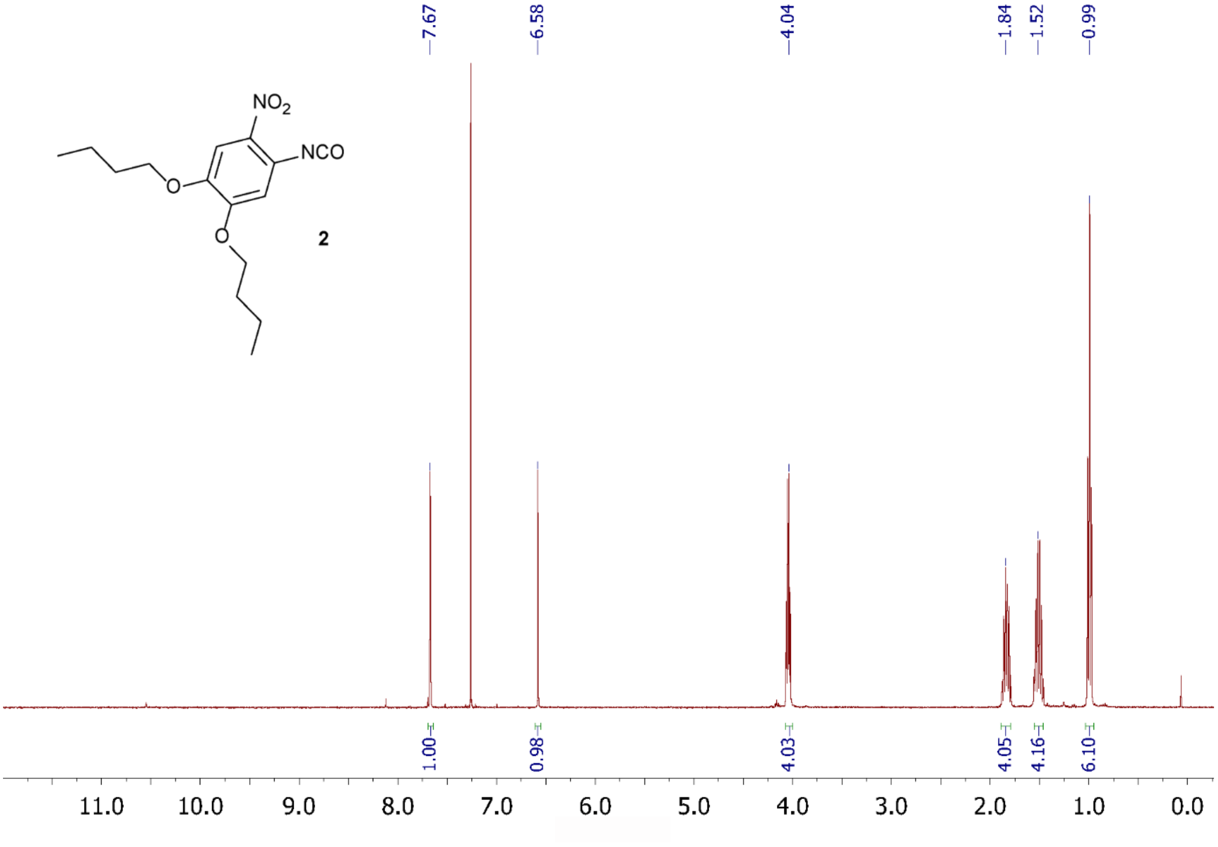

Figure S1. Compound **2**, ^{1}H NMR (400 MHz, chloroform-*d*).

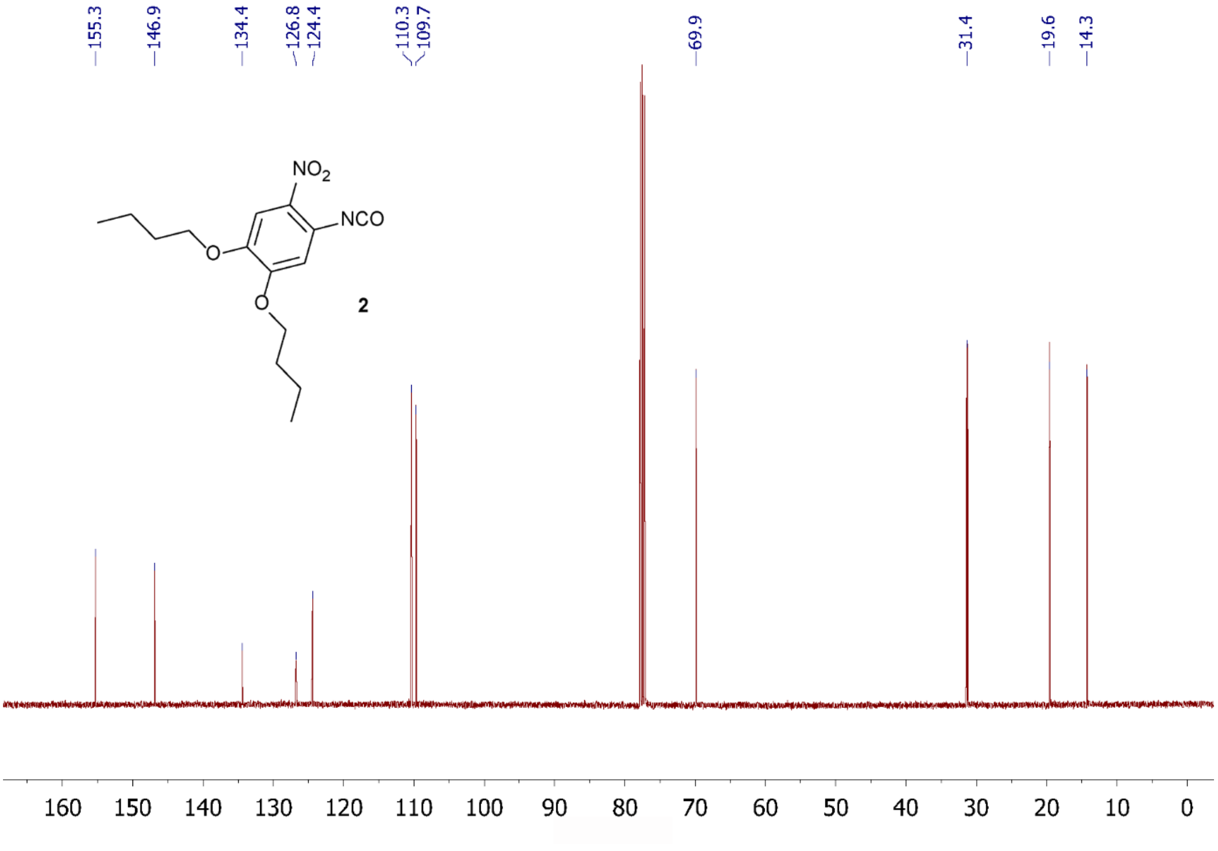

Figure S2. Compound **2**, ^{13}C NMR (101 MHz, chloroform-*d*).

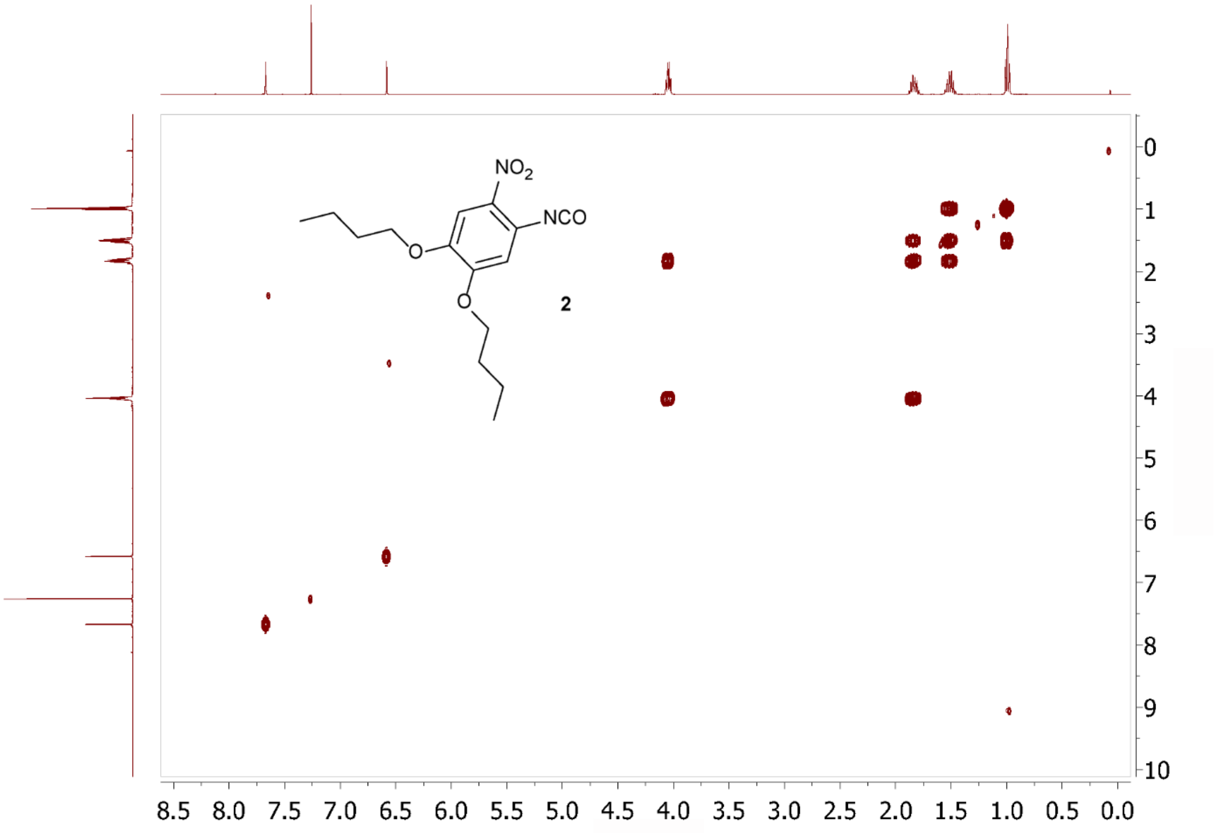

Figure S3. Compound **2**, ^{1}H-^{1}H COSY NMR (400 MHz, chloroform-*d*).

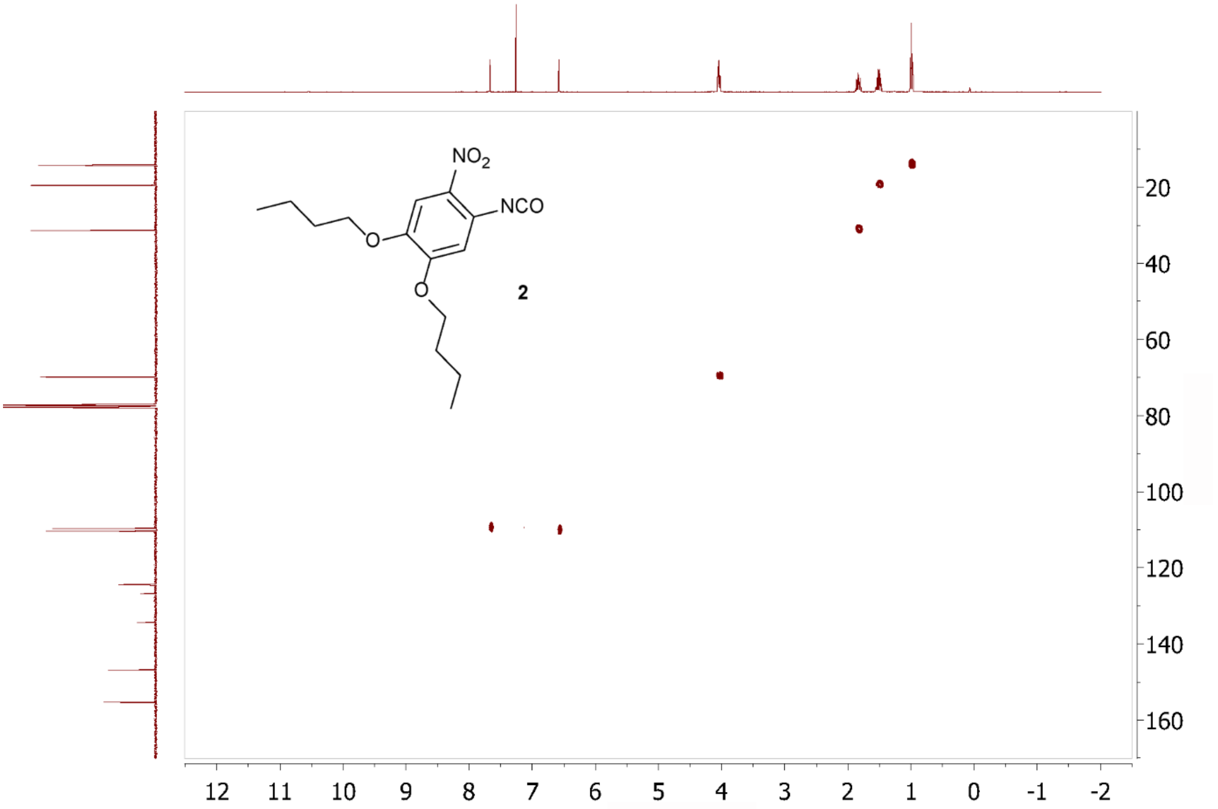

Figure S4. Compound **2**, ^{1}H-^{13}C HSQC NMR (400-101 MHz, chloroform-*d*).

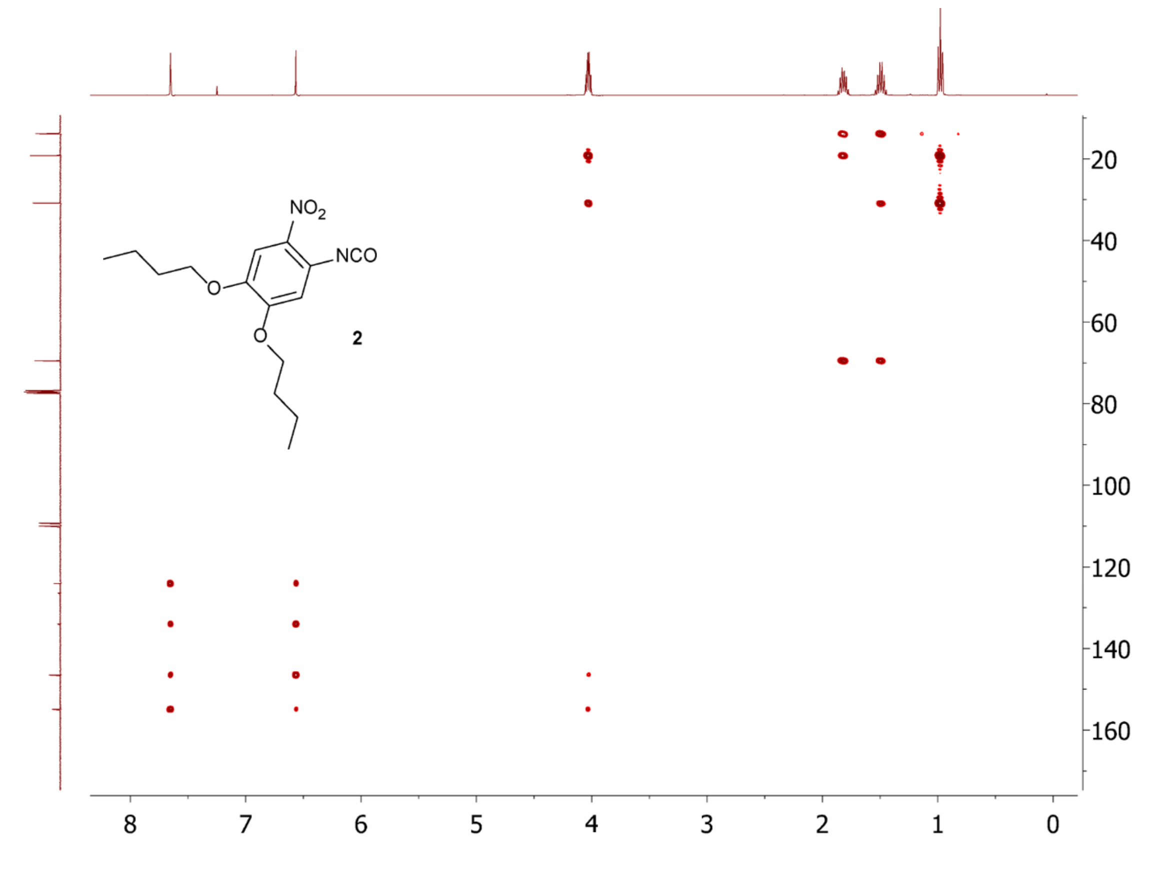

Figure S5. Compound **2**, ^{1}H-^{13}C HMBC NMR (400-101 MHz, chloroform-*d*).

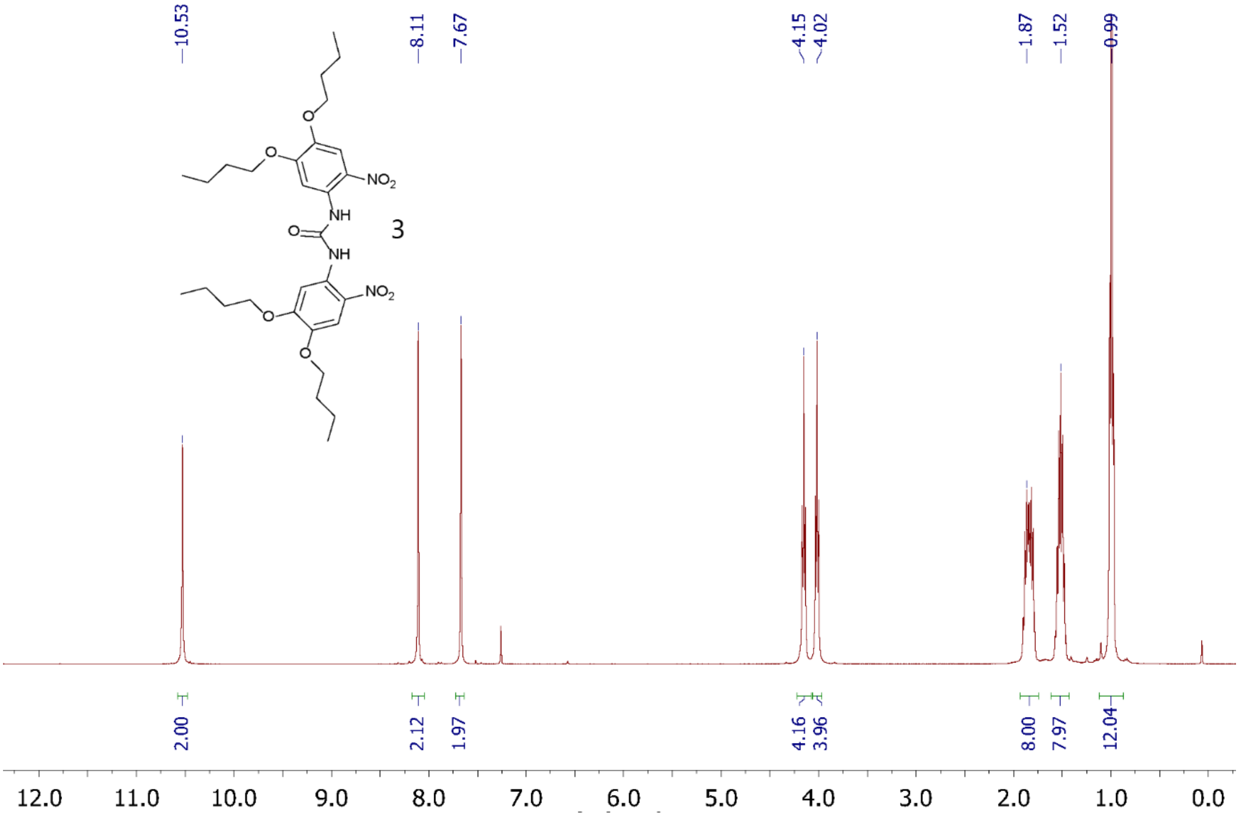

Figure S6. Compound **3**, ^{1}H NMR (400 MHz, chloroform-*d*).

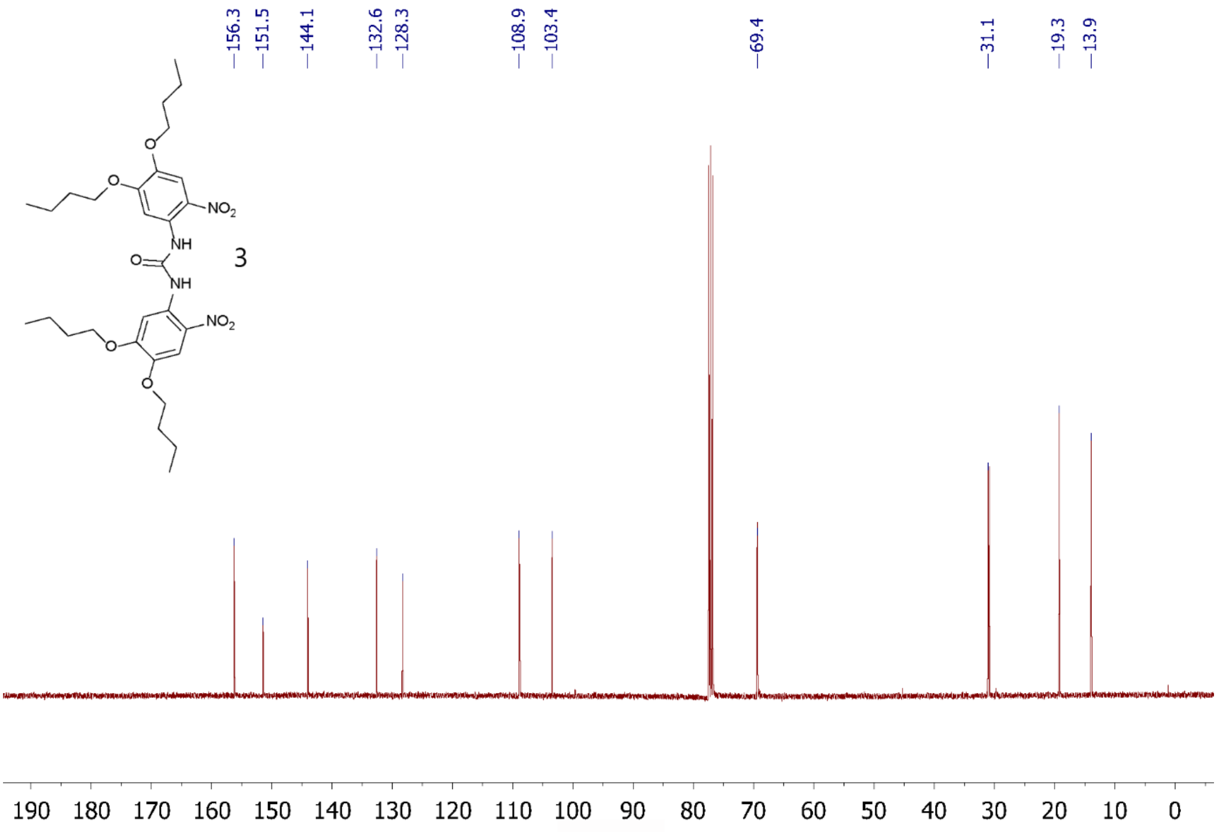

Figure S7. Compound **3**, ^{13}C NMR (101 MHz, chloroform-*d*).

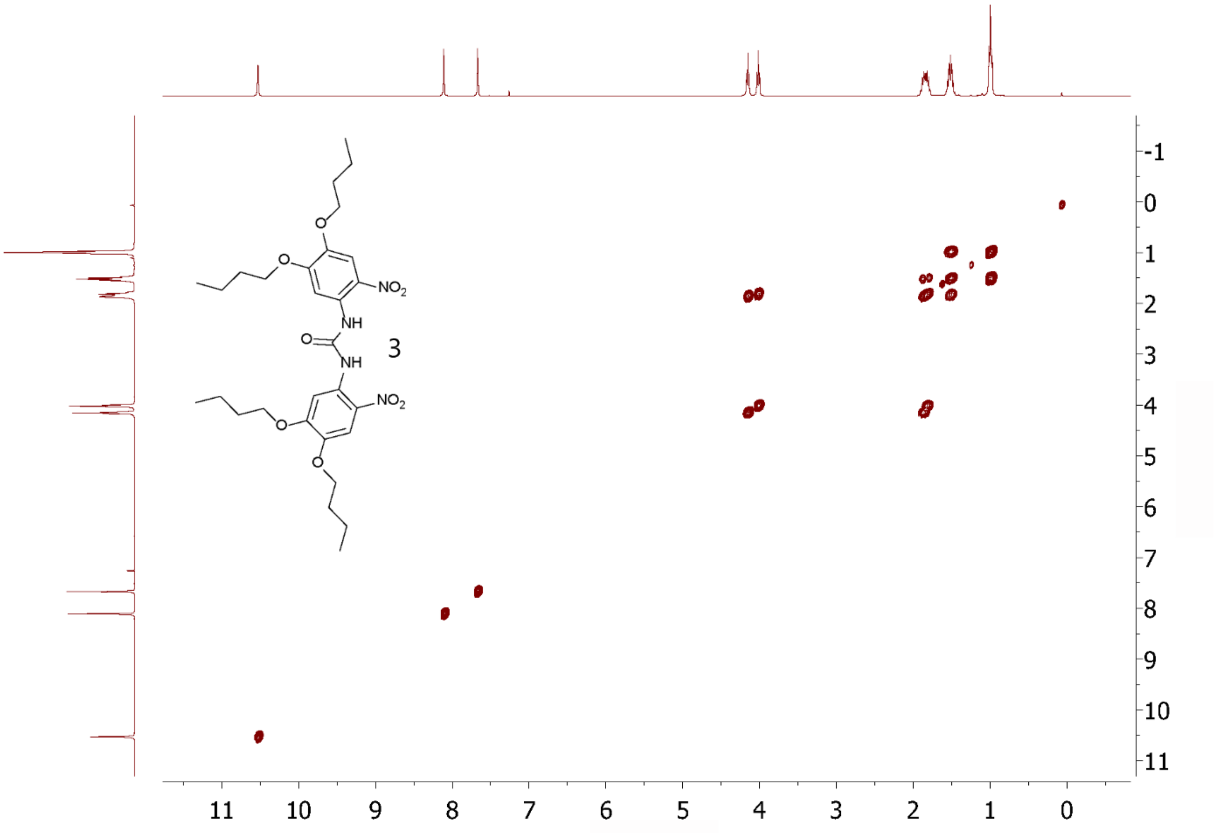

Figure S8. Compound **3**, ^{1}H-^{1}H COSY NMR (400 MHz, chloroform-*d*).

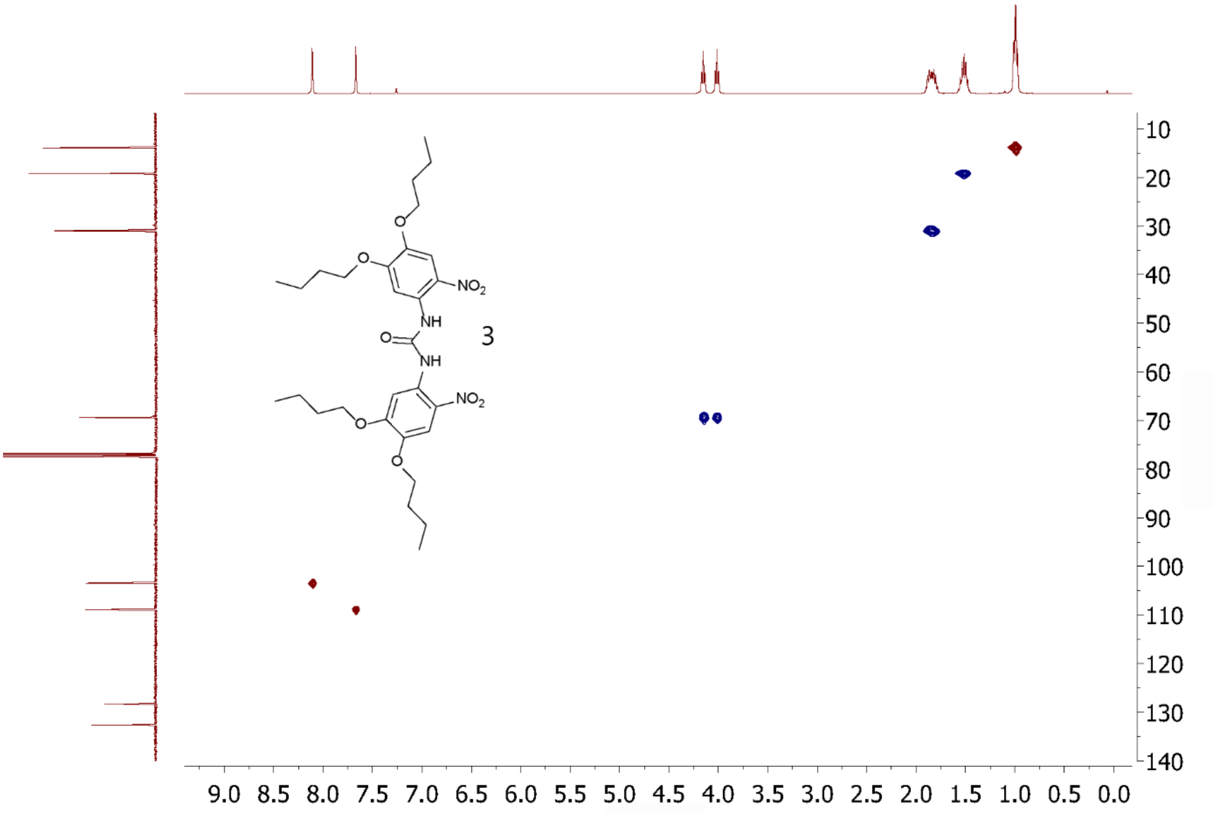

Figure S9. Compound **3**, ^{1}H-^{13}C HSQC NMR (400-101 MHz, chloroform-*d*).

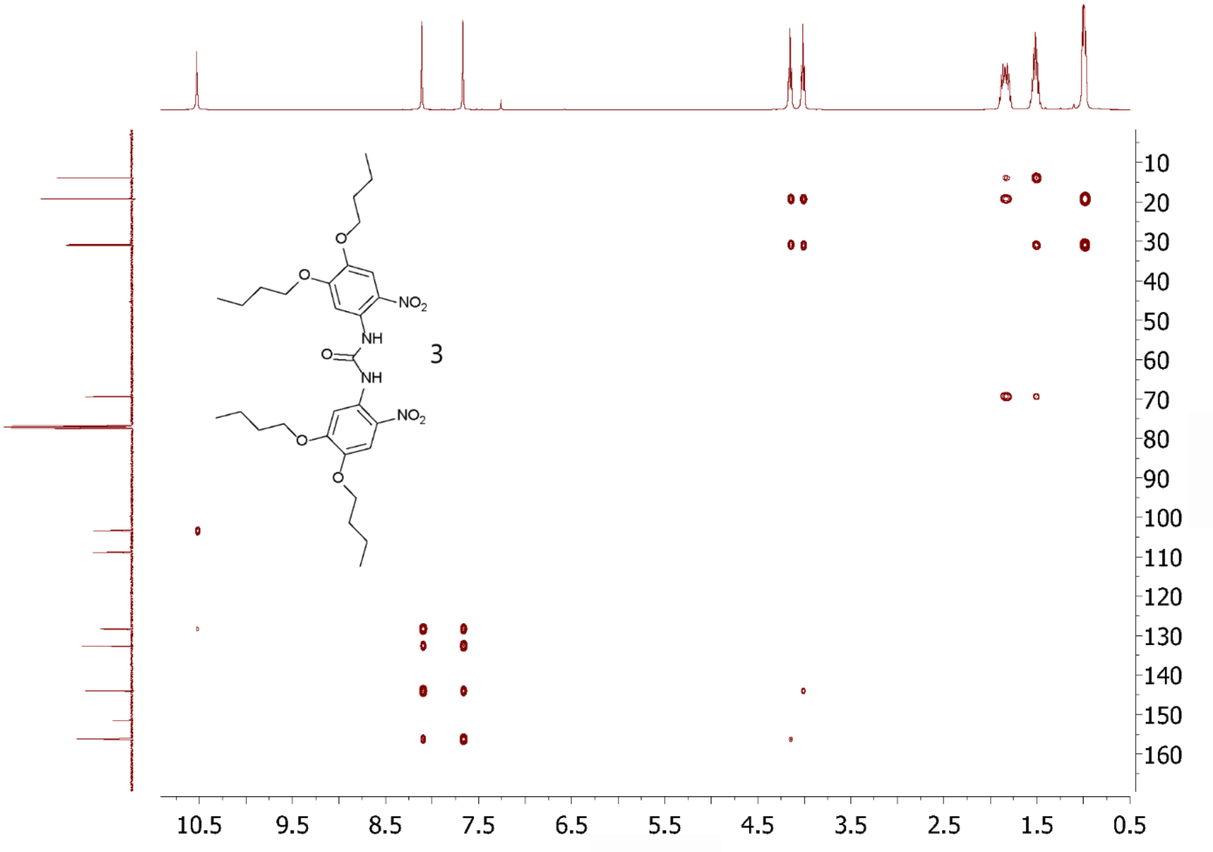

Figure S10. Compound **3**, ^{1}H-^{13}C HMBC NMR (400-101 MHz, chloroform-*d*).

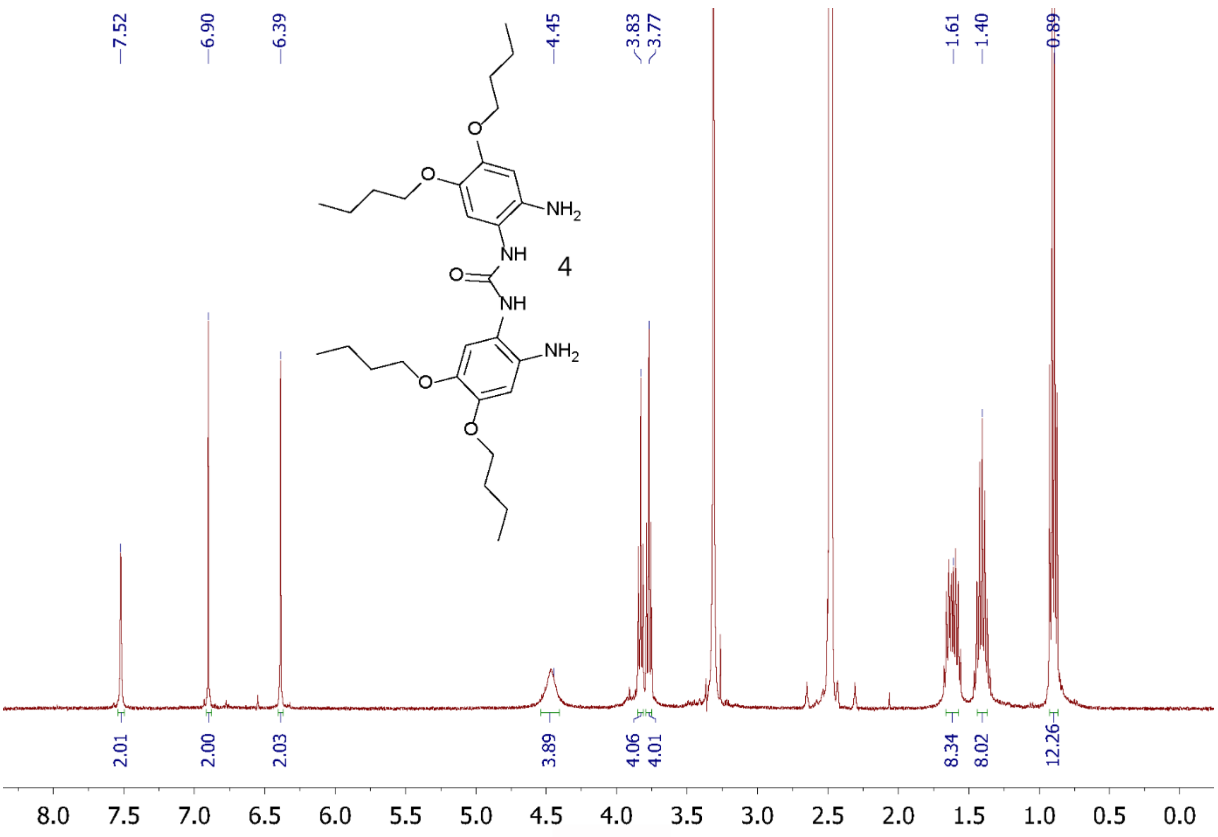

Figure S11. Compound **4**, ^{1}H NMR (400 MHz, DMSO-d_6).

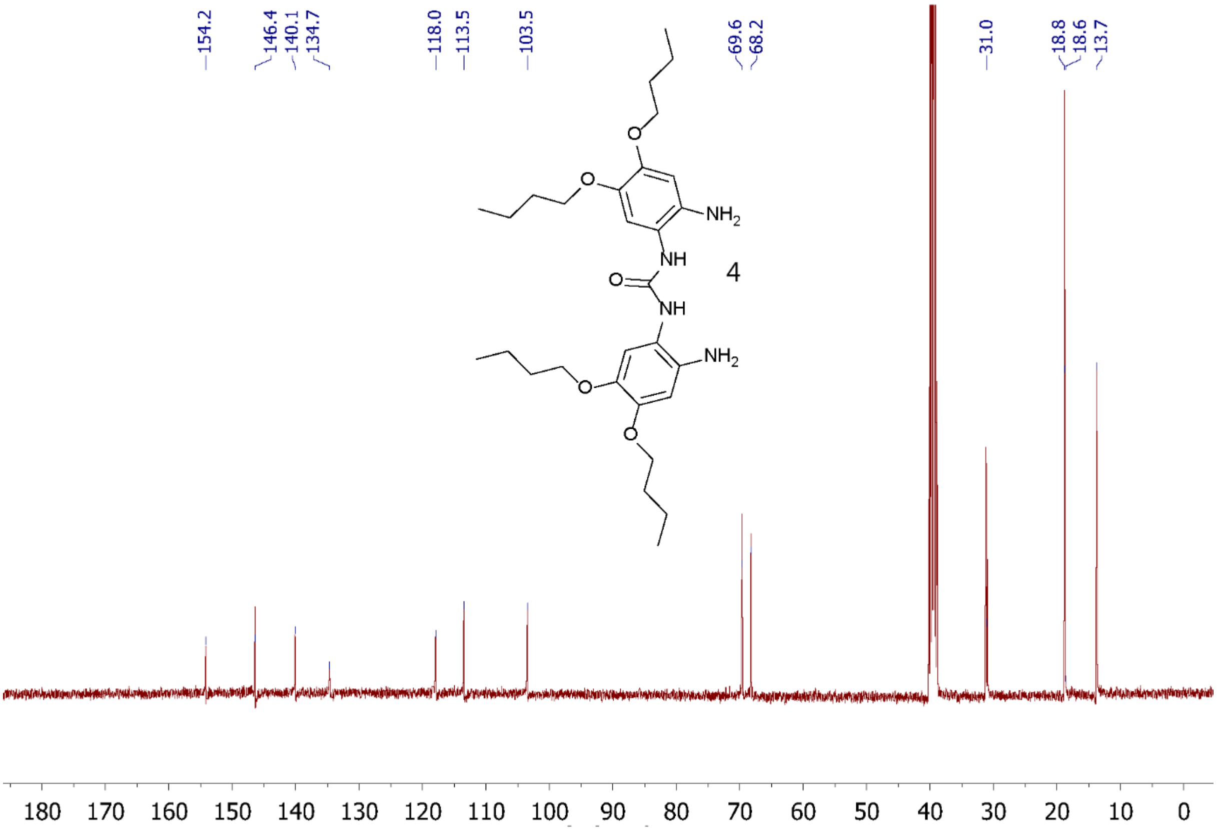

Figure S12. Compound **4**, ^{13}C NMR (101 MHz, DMSO- d_6).

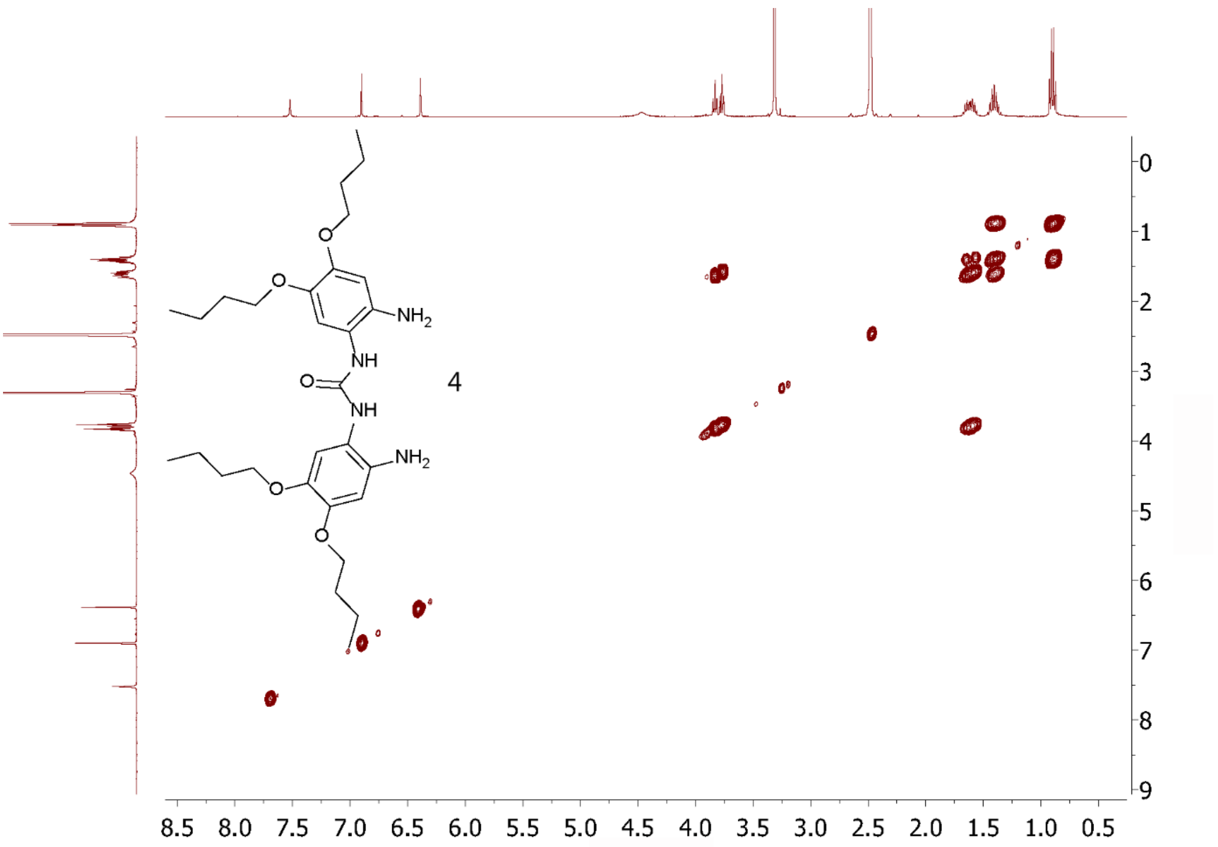

Figure S13. Compound **4**, ^{1}H- ^{1}H COSY NMR (404 MHz, DMSO- d_6).

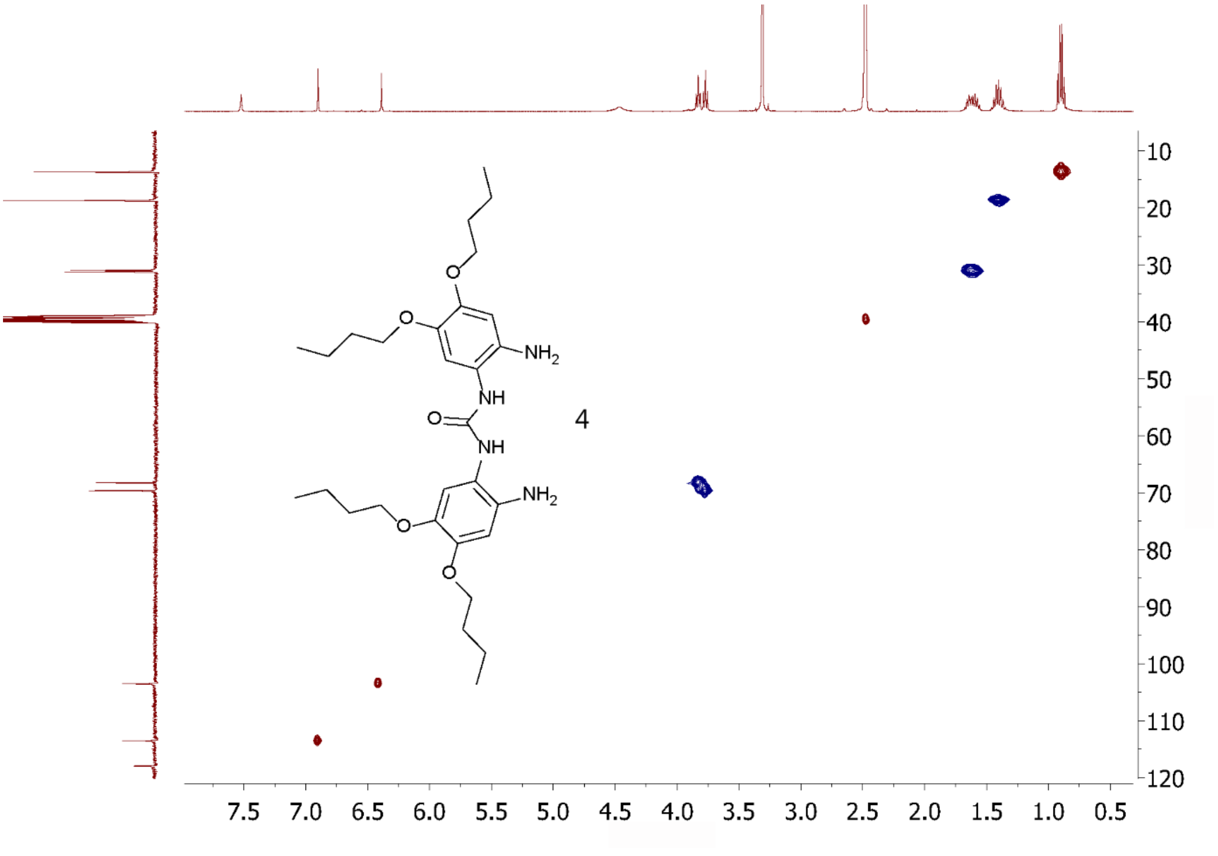

Figure S14. Compound **4**, ^{1}H- ^{13}C HSQC NMR (404-101 MHz, DMSO- d_6).

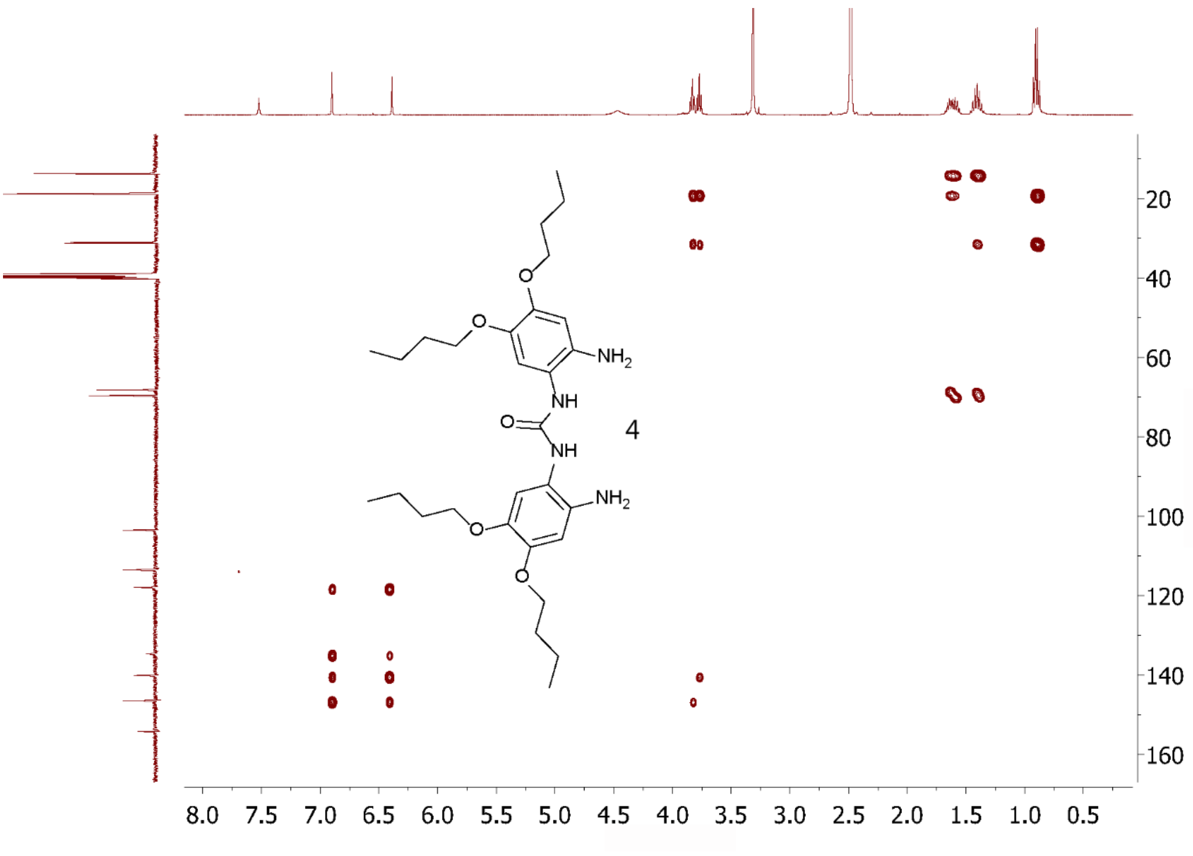

Figure S15. Compound **4**, ${}^{1}H$- ${}^{13}C$ HMBC NMR (404-101 MHz, DMSO- d_6).

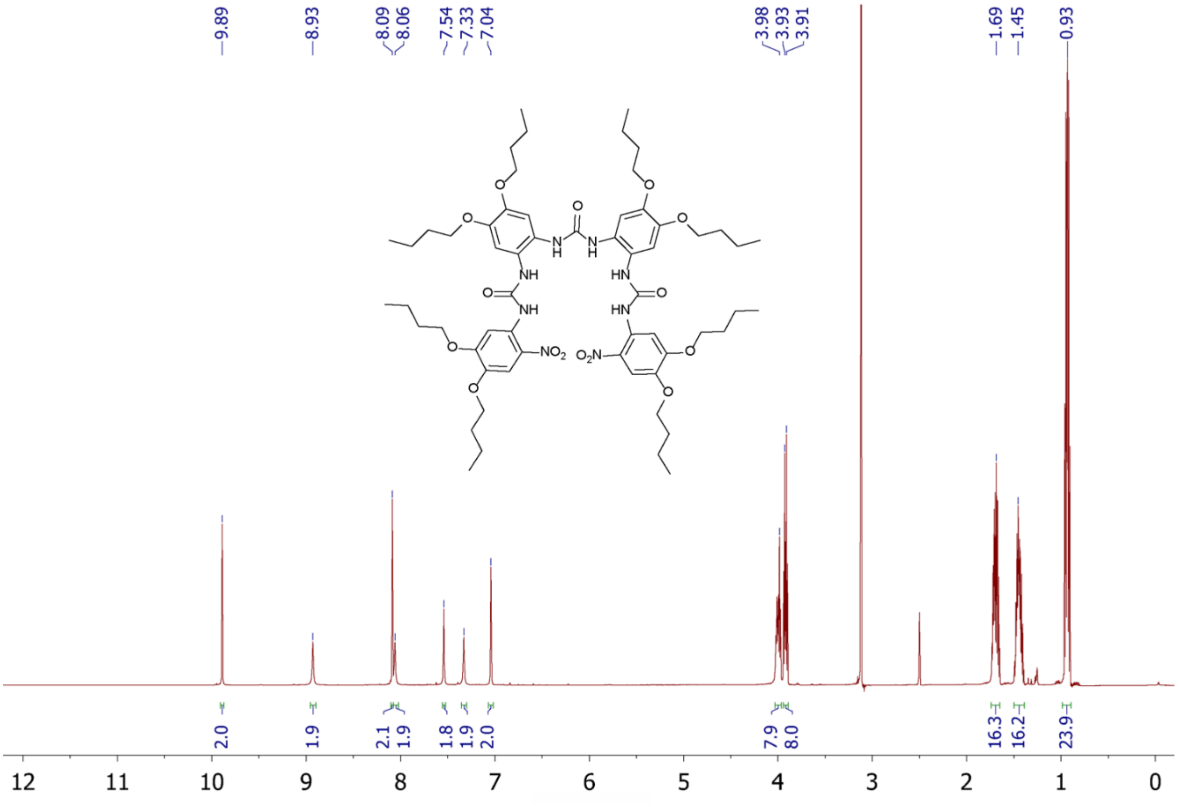

Figure S16. Compound **5**, ${}^{1}H$ NMR (404 MHz, DMSO- d_6, 70° C).

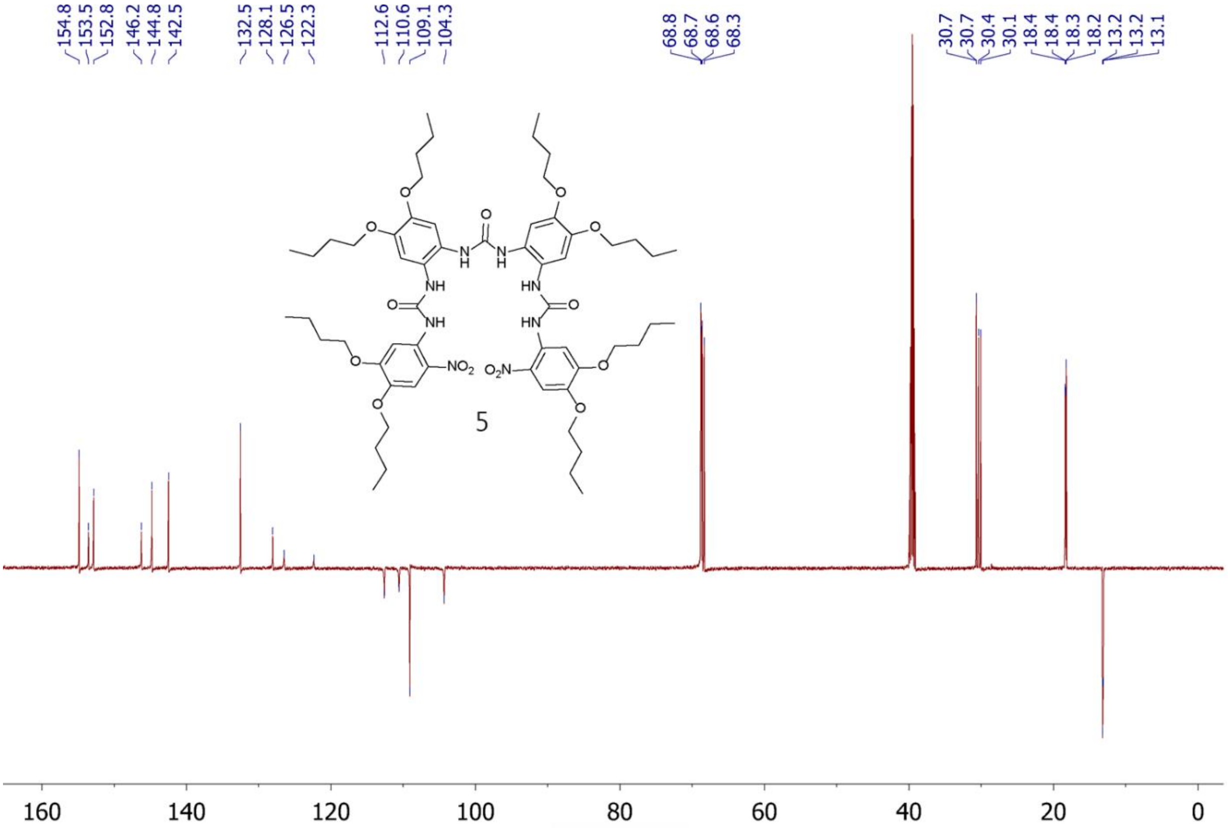

Figure S17. Compound **5**, ^{13}C APT NMR (101 MHz, DMSO- d_6, 70° C).

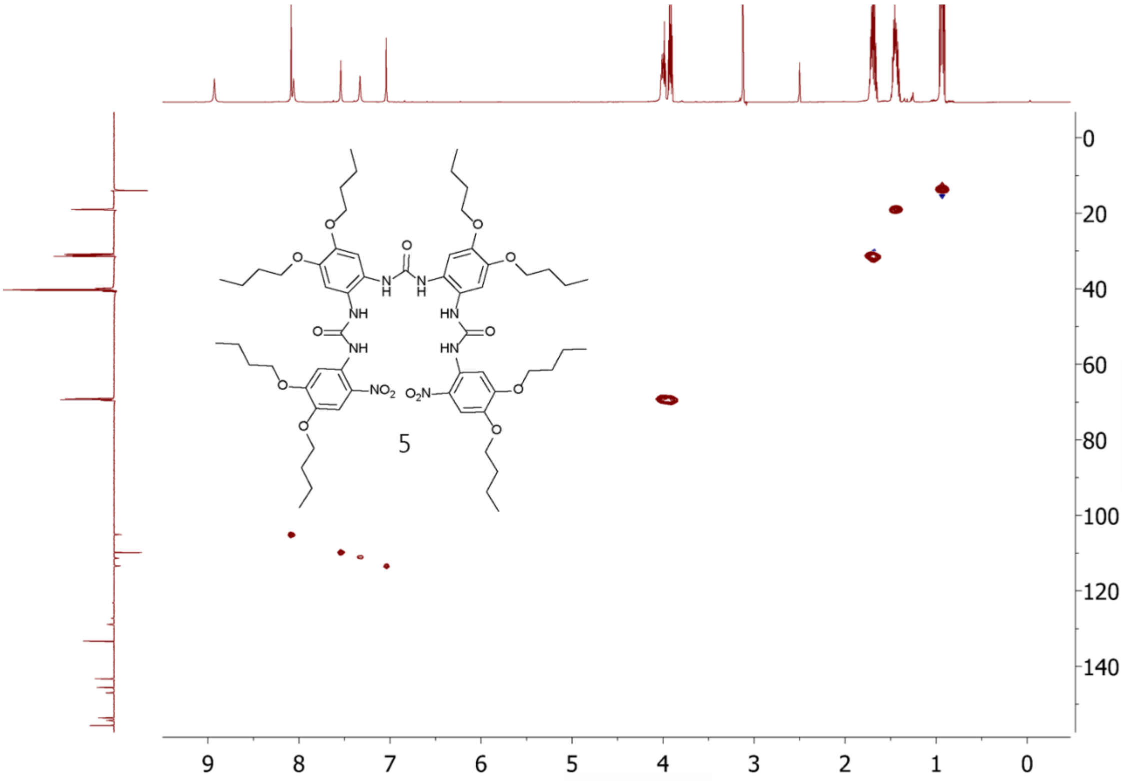

Figure S18. Compound **5**, ^{1}H- ^{13}C HSQC NMR (404-101 MHz, DMSO- d_6, 70° C).

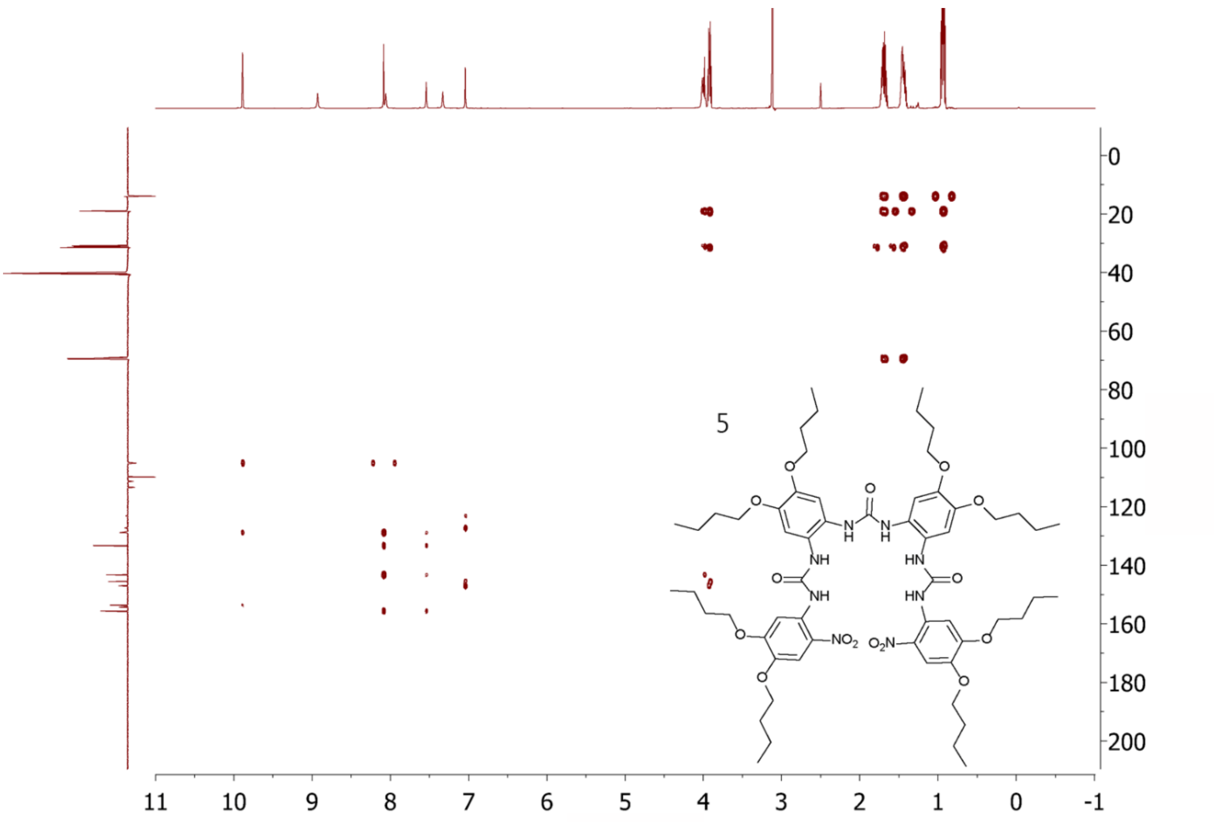

Figure S19. Compound **5**, ^{1}H- ^{13}C HMBC NMR (404-101 MHz, DMSO- d_6, 70° C).

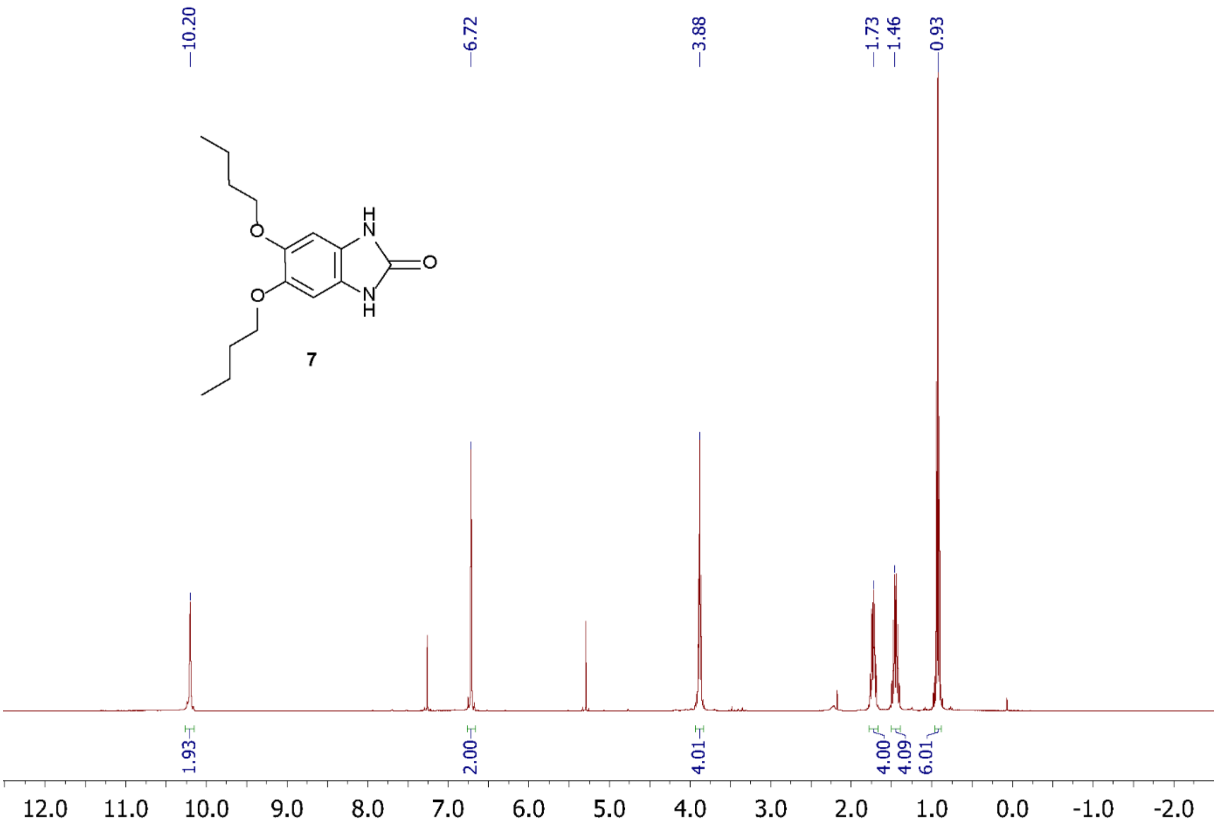

Figure S20. Compound **7**, ^{1}H NMR (404 MHz, chloroform-*d*).

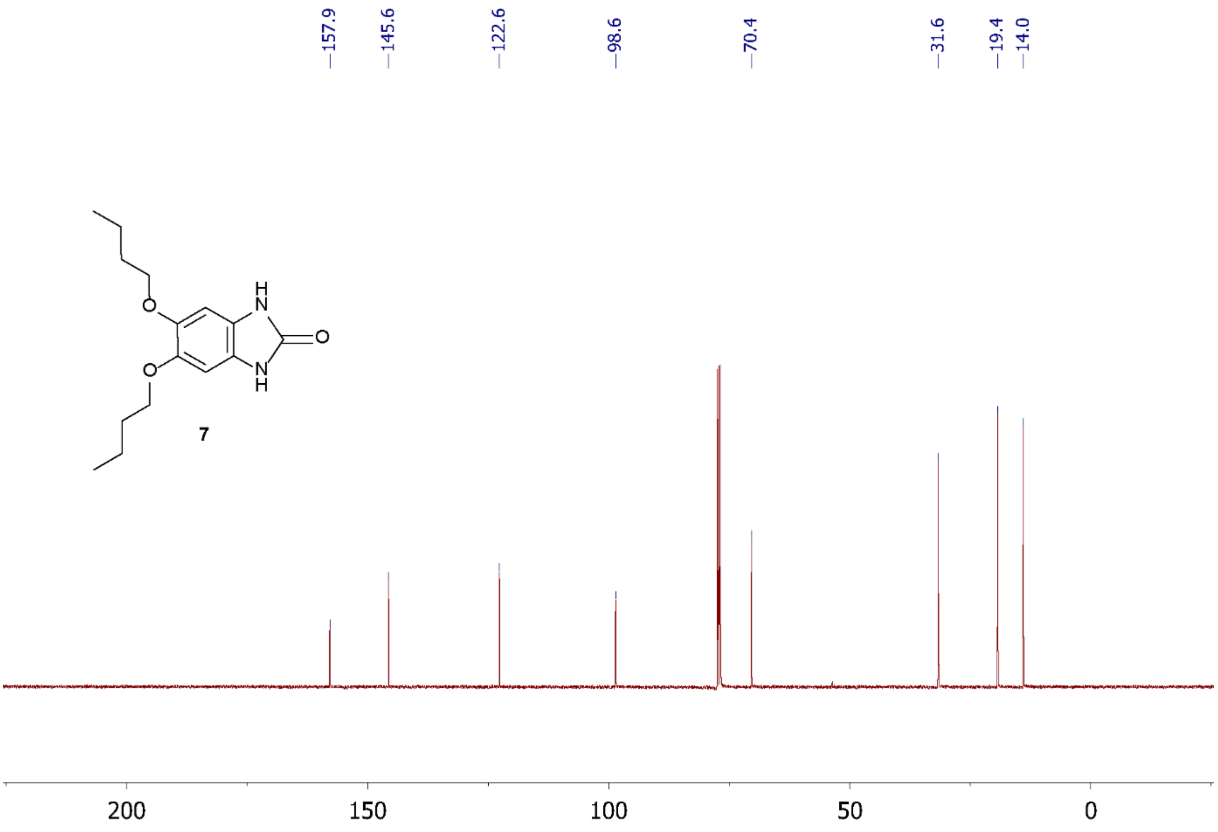

Figure S21. Compound **7**, ^{13}C NMR (101 MHz, chloroform-*d*).

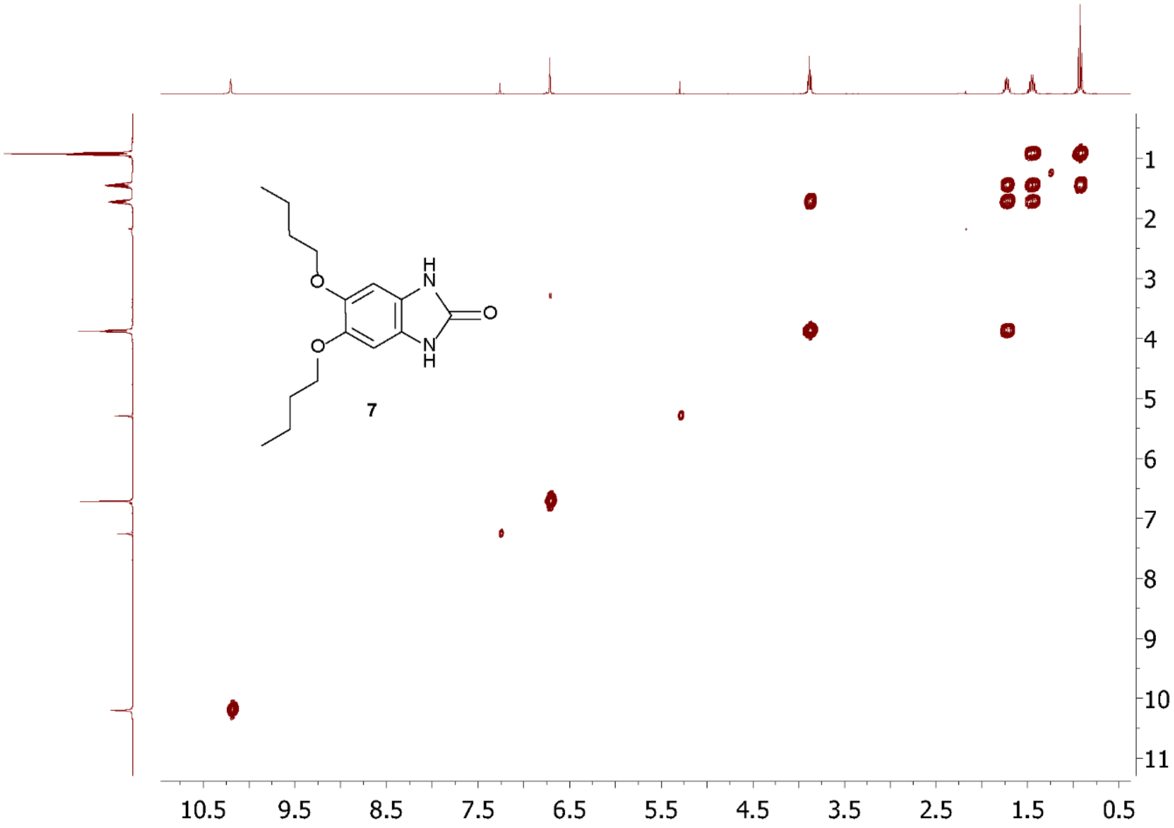

Figure S22. Compound **7**, ^{1}H- ^{1}H COSY NMR (404 MHz, chloroform-*d*).

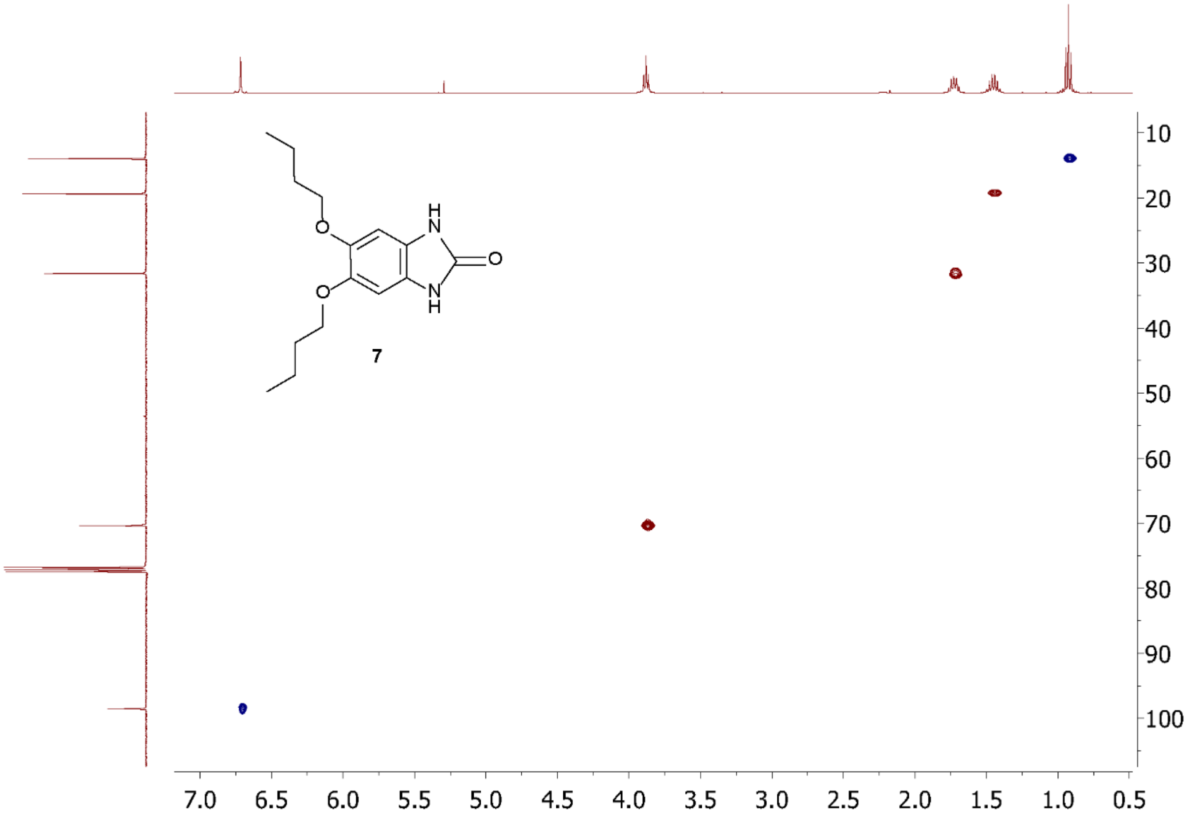

Figure S23. Compound **7**, ^{1}H-^{13}C HSQC NMR (404-101 MHz, chloroform-*d*).

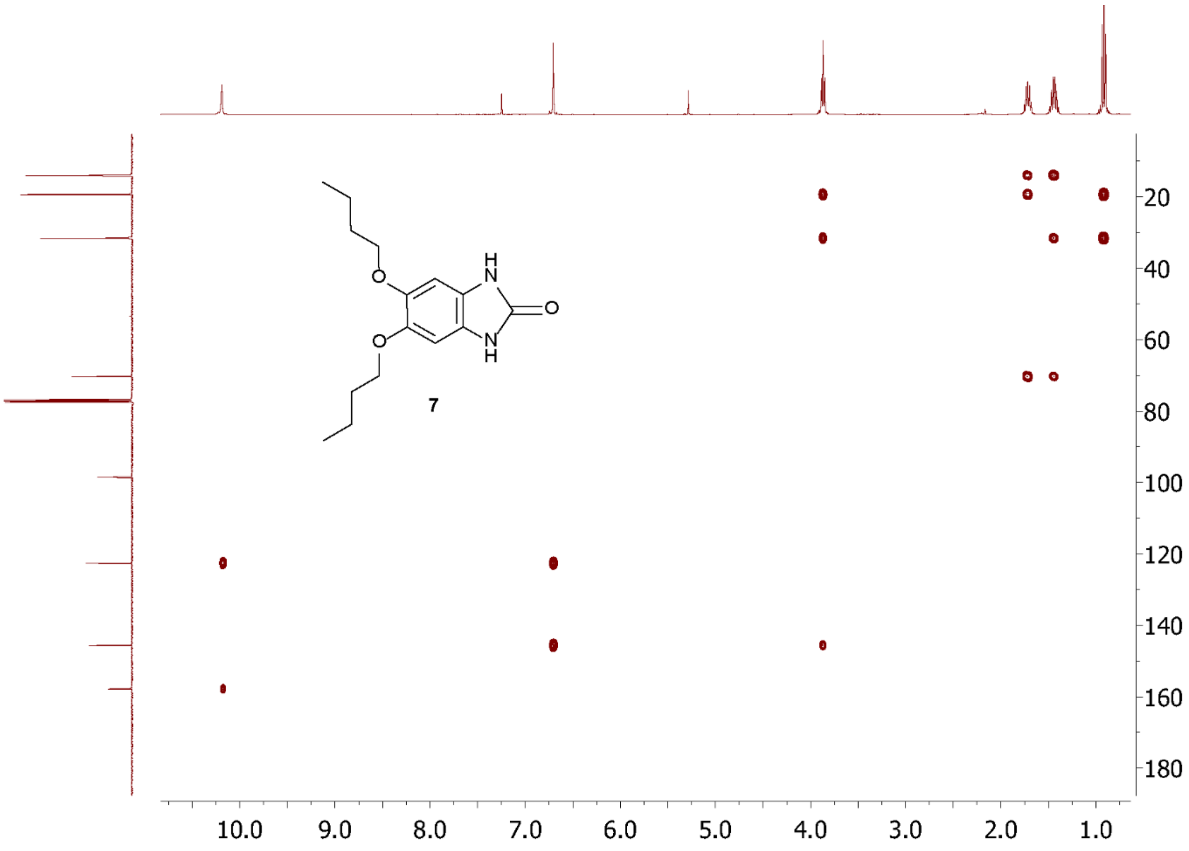

Figure S24. Compound **7**, ^{1}H-^{13}C HMBC NMR (404-101 MHz, chloroform-*d*).

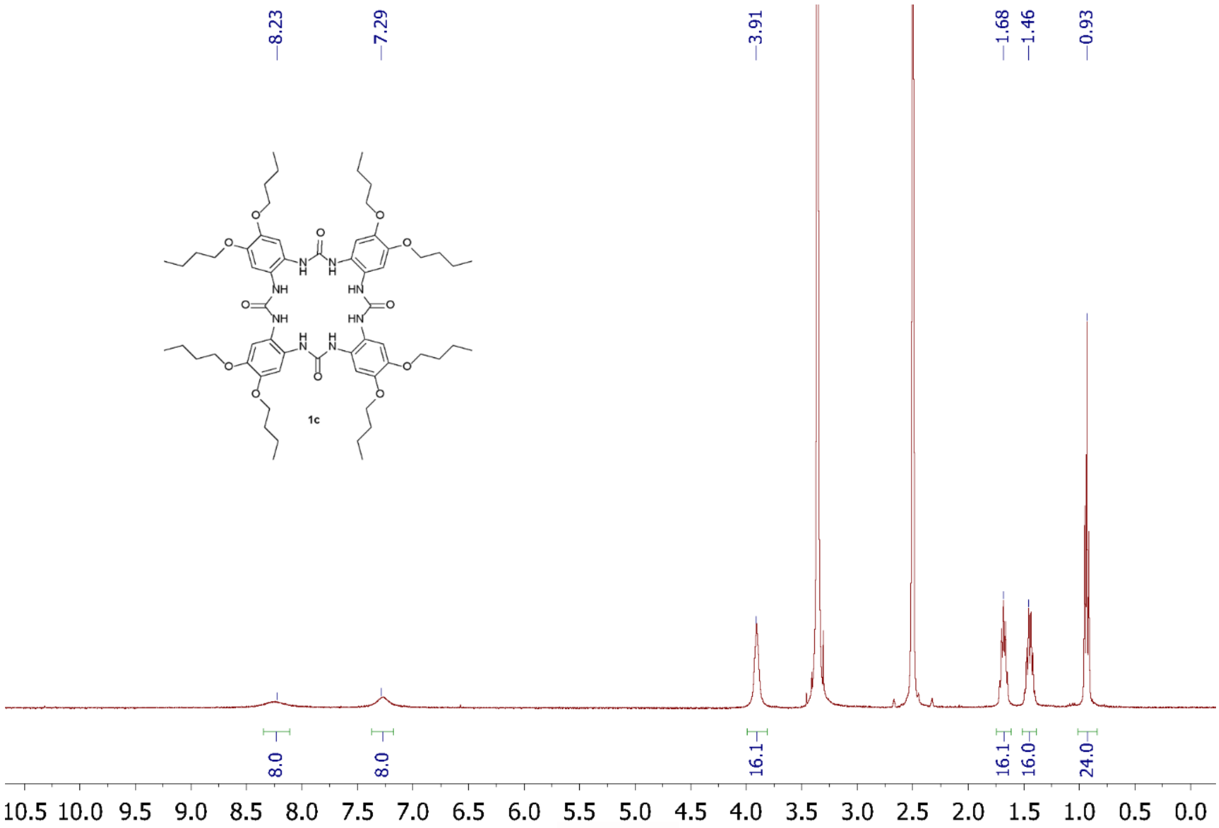

Figure S25. Compound **1c**, ^{1}H NMR (404 MHz, DMSO- d_6, 25° C).

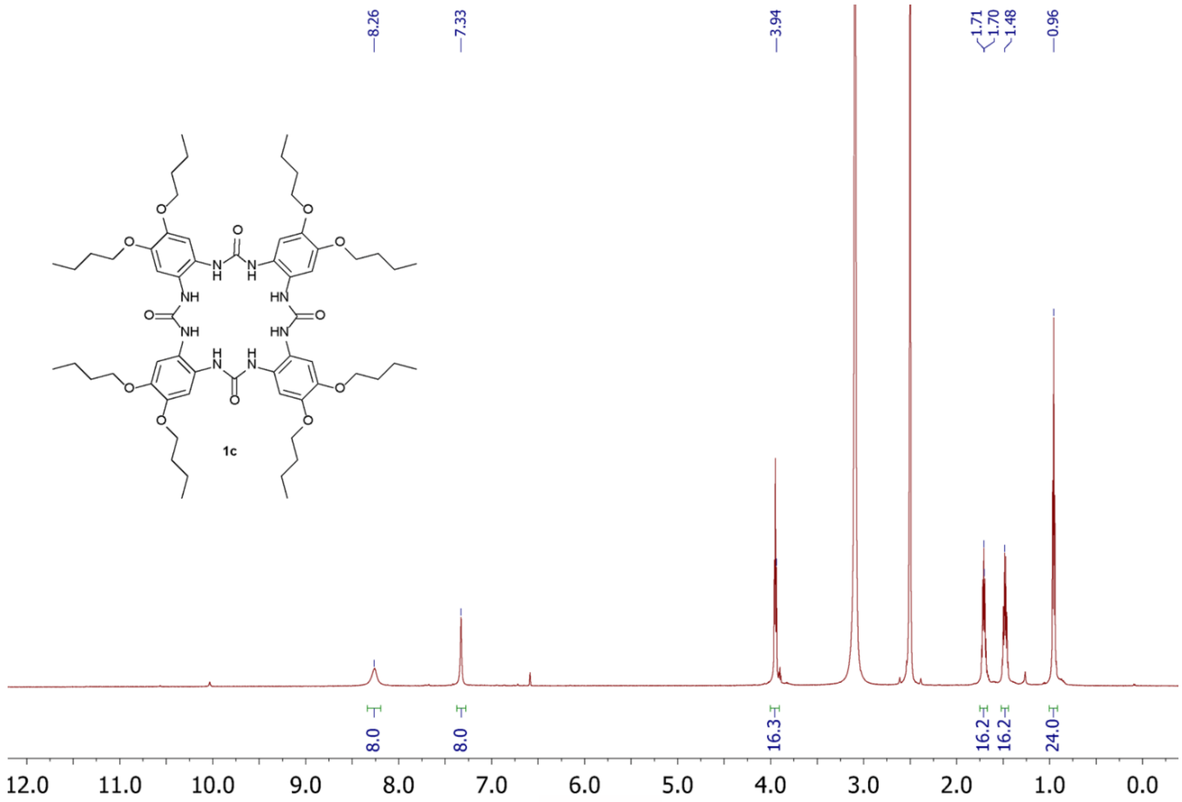

Figure S26. Compound **1c**, ^{1}H NMR (404 MHz, DMSO- d_6, 70° C).

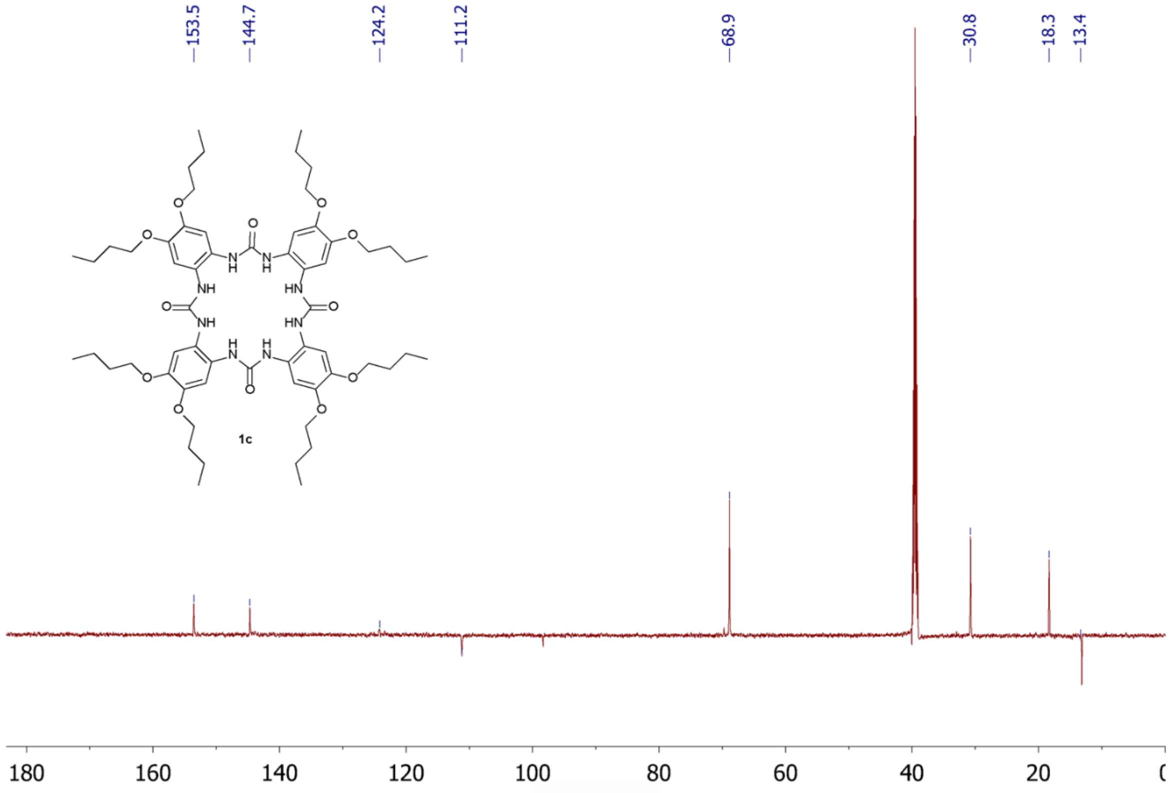

Figure S27. Compound **1c**, ^{13}C APT NMR (101 MHz, DMSO- d_6, 70° C).

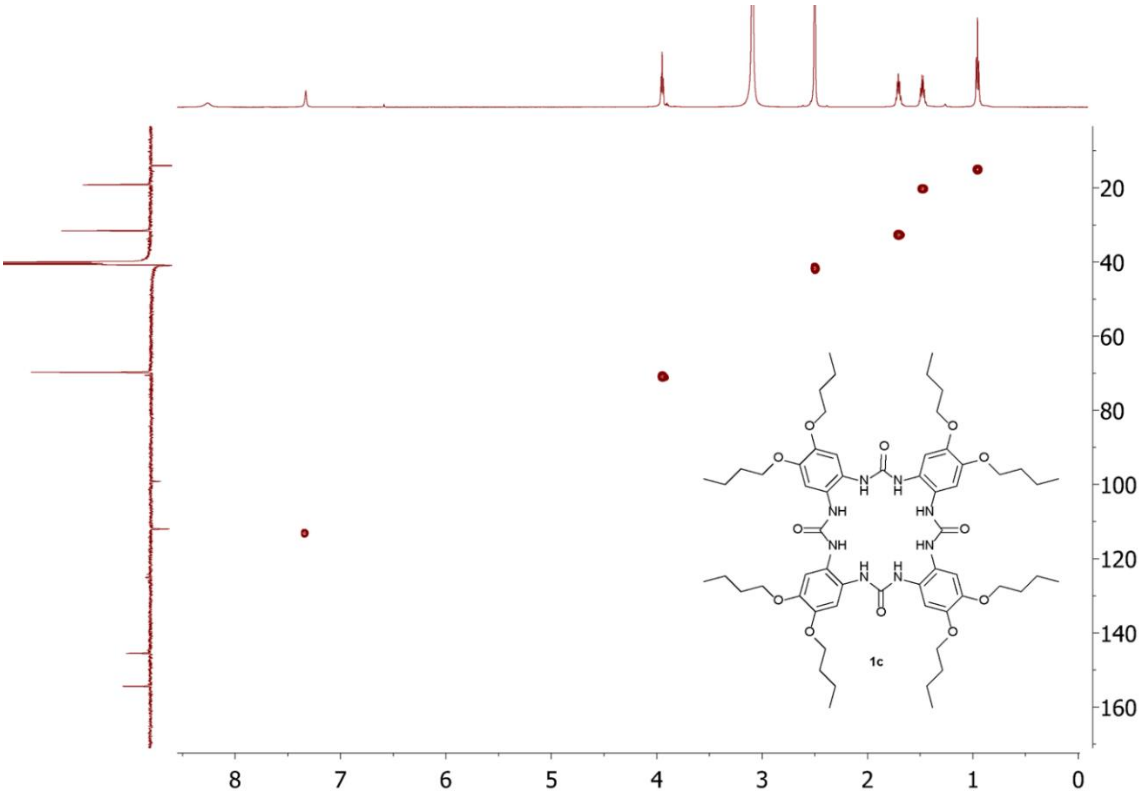

Figure S28. Compound **1c**, ^{1}H- ^{13}C HSQC NMR (404-101 MHz, DMSO- d_6, 70° C).

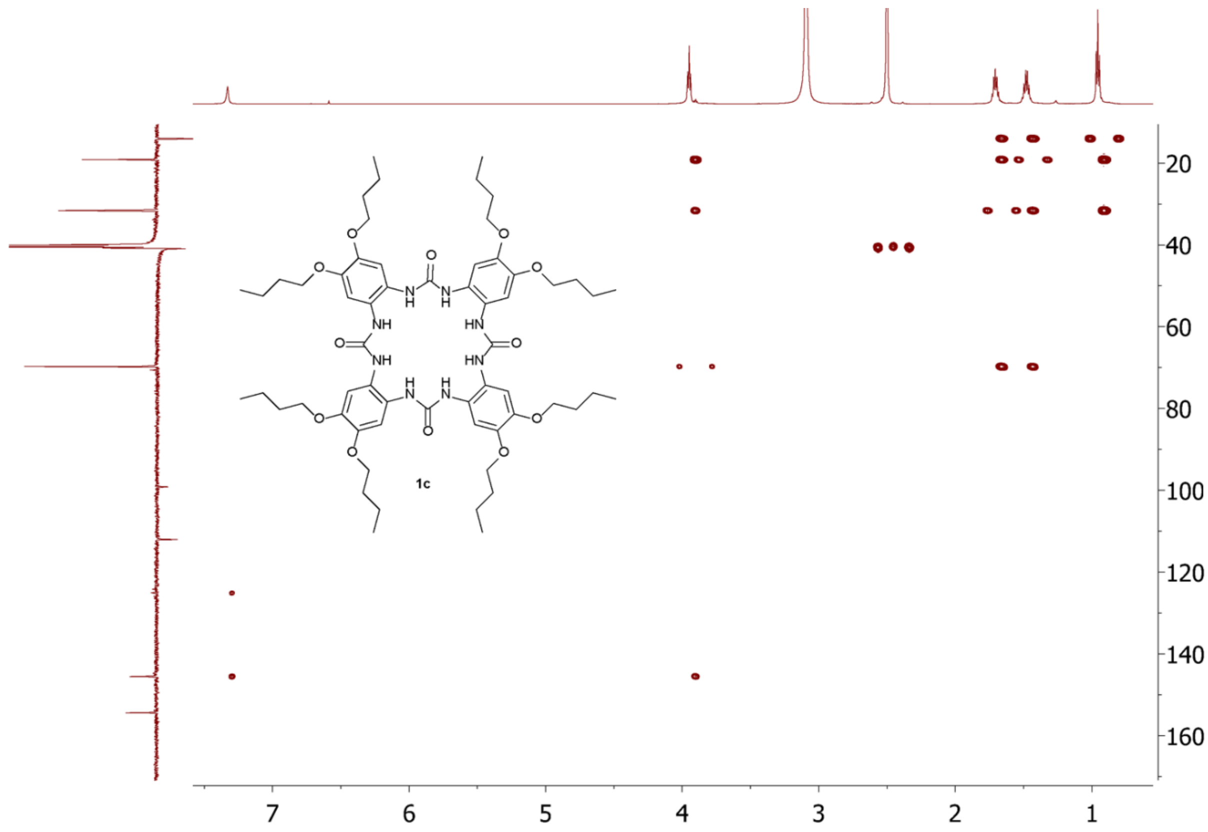

Figure S29. Compound **1c**, ^{1}H- ^{13}C HMBC NMR (404-101 MHz, DMSO- *d*$_6$, 70° C).

NMR titrations

c 1c [M]	c anion [M]	$\Delta\delta$ NH [Hz]
0.002713	0	0
0.002713	0.000677	31.68
0.002713	0.001955	70.53
0.002713	0.003139	95.44
0.002713	0.004241	109.53
0.002713	0.005755	123.39
0.002713	0.007969	136.28
0.002713	0.009866	144.02
0.002713	0.011511	149.13
0.002713	0.01295	153
0.002713	0.015348	158.96
0.002713	0.016823	162.43
0.002713	0.05791	193.72

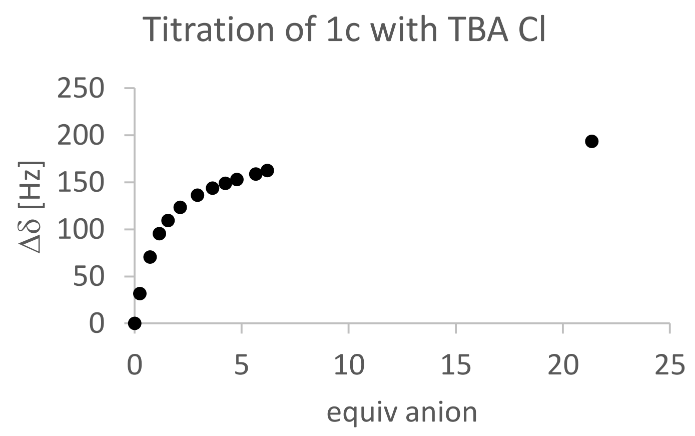

Figure S30. Compound **1c**, ^{1}H NMR titration with TBA chloride, $\Delta\delta$ NH, 25°C, DMSO- *d*$_6$.

c 1c [M]	c anion [M]	Δδ NH [Hz]
0.00265	0	0
0.00265	0.00032	4.9
0.00265	0.000925	6.6
0.00265	0.001485	12.8
0.00265	0.002006	13.6
0.00265	0.002723	18.9
0.00265	0.00377	22.9
0.00265	0.004667	24.1
0.00265	0.005445	26.7
0.00265	0.006126	29.1
0.00265	0.00726	33.3
0.00265	0.007915	36.6
0.00265	0.03267	55.1

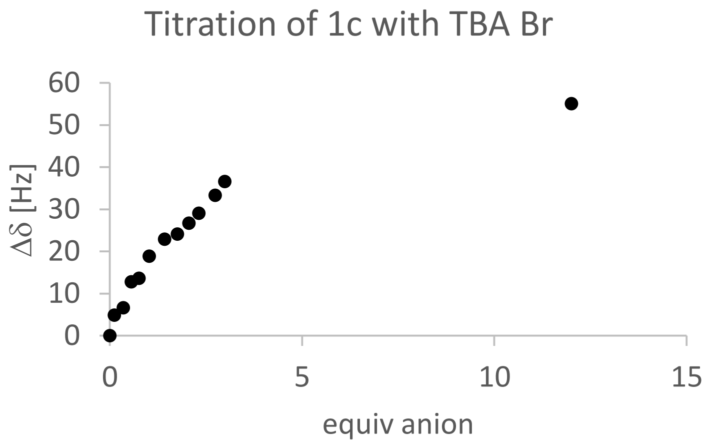

Figure S31. Compound **1c**, ^{1}H NMR titration with TBA bromide, Δδ NH, 25°C, DMSO- d_6.

c 1c [M]	c anion [M]	Δδ NH [Hz]
0.002713	0	0
0.002713	0.0006795	60.1
0.002713	0.0019615	148.3
0.002713	0.0031503	185.17
0.002713	0.0042557	205.98
0.002713	0.0057756	221.47
0.002713	0.007997	274.13
0.002713	0.009901	258.37
0.002713	0.0115512	272.72
0.002713	0.012995	283.16
0.002713	0.0154015	299.2
0.002713	0.016937	308.14
0.002713	0.0346535	383.45

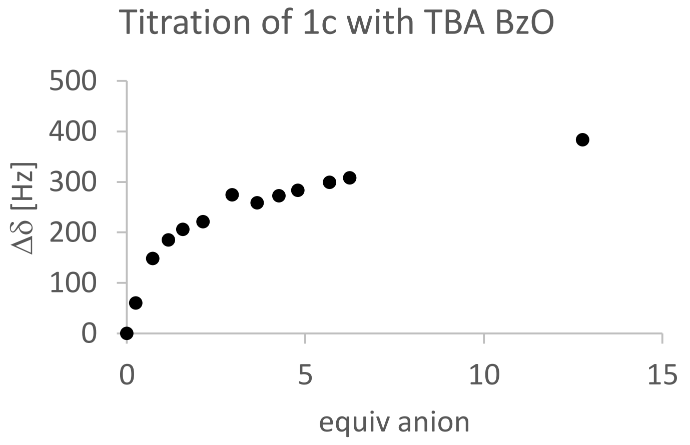

Figure S32. Compound **1c**, ^{1}H NMR titration with TBA benzoate, Δδ NH, 25°C, DMSO- d_6.

c 1c [M]	c anion [M]	Δδ [Hz] NHα	Δδ [Hz] NHβ
0.00265	0	0	0
0.00265	0.000677	75.66	75.66
0.00265	0.001954	238.69	238.69
0.00265	0.003139	318.28	318.28
0.00265	0.00424	354.71	527.93
0.00265	0.005754	356.39	649.13
0.00265	0.007968	355.02	748.83
0.00265	0.009865	353.92	797.62
0.00265	0.011509	354.4	821.18
0.00265	0.012947	354.52	826.47
0.00265	0.015345	351.74	832.59
0.00265	0.016839	360.14	827.59

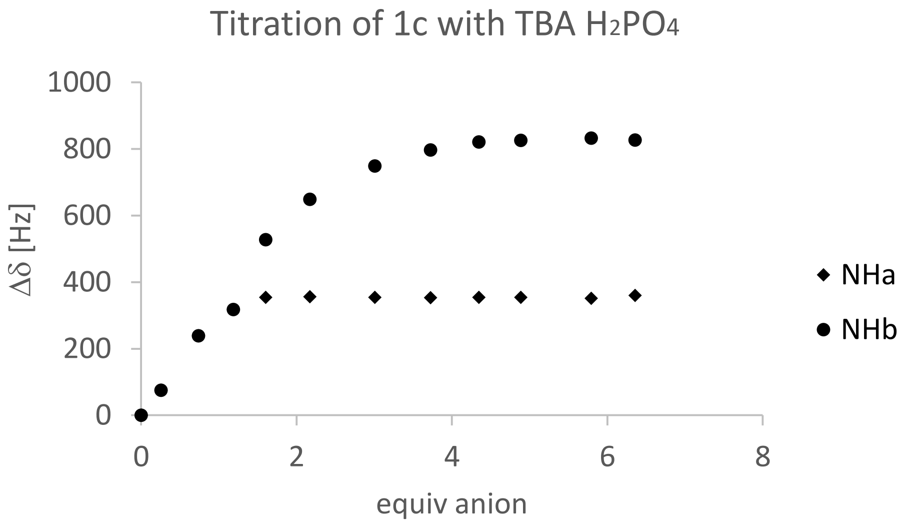

Figure S33. Compound **1c**, ^{1}H NMR titration with TBA dihydrogenphosphate, Δδ NH, 25°C, DMSO- *d*$_6$.

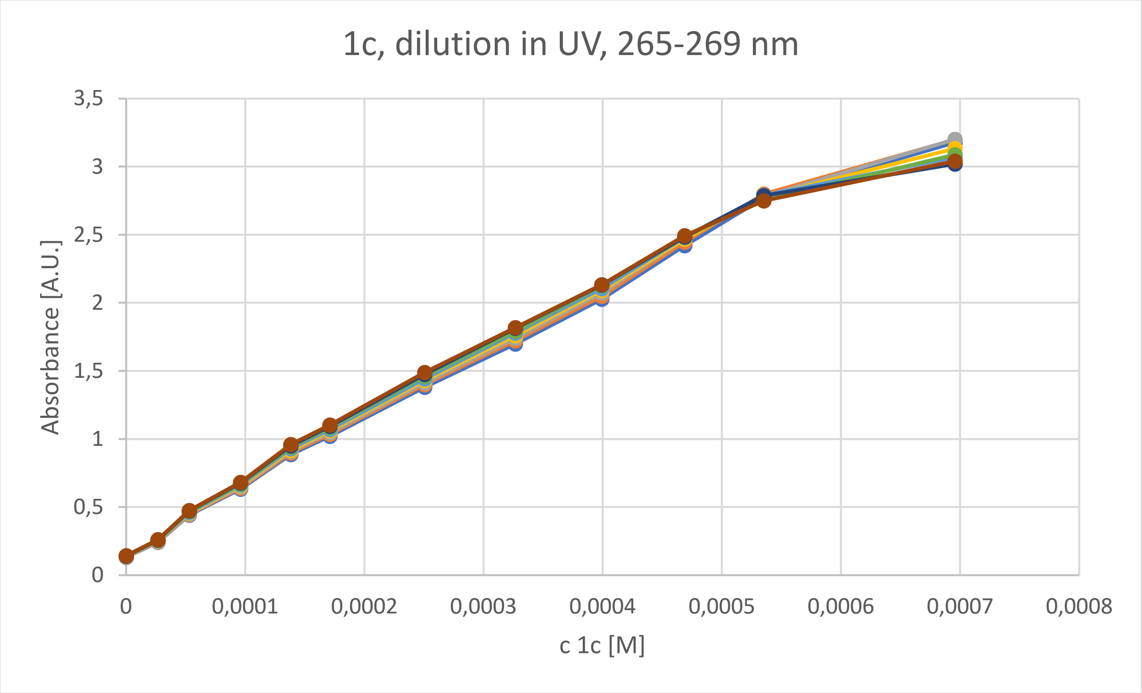

Figure S34. Compound **1c**, UV-Vis, DMSO, 25°C. Changes in absorbation with concentration of 1c at 265-269 nm.

UV-Vis titrations

c 1c [M]	c anion [M]	267 nm	267.5 nm	268 nm	268.5 nm	270 nm
4.04E-05	0	0.402298	0.407899	0.410829	0.416505	0.421027
4.02E-05	9.42E-05	0.39939	0.404869	0.409087	0.41469	0.419573
4E-05	0.000188	0.408151	0.412499	0.417262	0.420731	0.426052
3.98E-05	0.00028	0.404981	0.409155	0.414408	0.41924	0.423754
3.93E-05	0.000507	0.406641	0.411461	0.415219	0.42105	0.425029
3.83E-05	0.000988	0.410869	0.414647	0.419976	0.424229	0.429458
3.74E-05	0.001403	0.404245	0.409118	0.412076	0.417852	0.421248
3.66E-05	0.0018	0.398033	0.402523	0.406991	0.411426	0.416156
3.58E-05	0.002179	0.392951	0.396581	0.401829	0.406859	0.411926
3.5E-05	0.002542	0.380049	0.384687	0.387595	0.393655	0.396877
3.36E-05	0.003223	0.365346	0.368768	0.373554	0.376704	0.381651
3.22E-05	0.003849	0.347711	0.350947	0.355029	0.359656	0.363327
2.98E-05	0.004963	0.331993	0.335844	0.338312	0.342894	0.346781
2.83E-05	0.005696	0.313081	0.316446	0.319055	0.323811	0.326703

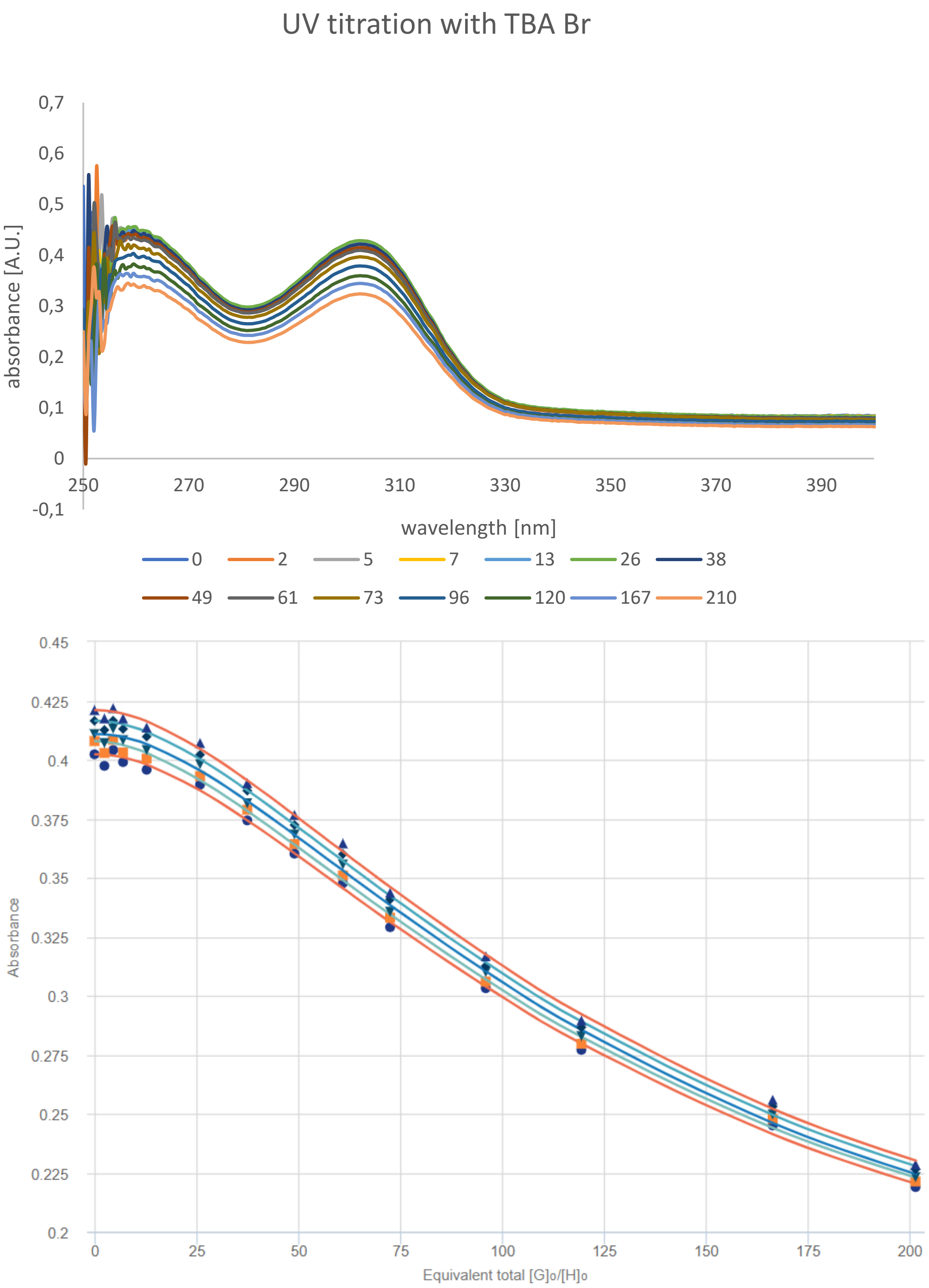

Figure S35. Compound **1c**, UV-Vis titration with TBA bromide, 25°C, DMSO, , with depicted equivalents of aniont (top), .Bindfit calculated fit for selecte wavelengths (bottom).

.

c 1c [M]	c anion [M]	267 nm	267.5 nm	268 nm	268.5 nm	270 nm
4.04E-05	0.00E+00	0.400155	0.408394	0.411693	0.41791	0.421325
4.02E-05	7.80E-05	0.442246	0.451021	0.455067	0.460764	0.45427
4.00E-05	1.55E-04	0.501871	0.509604	0.513745	0.519353	0.522399
3.98E-05	2.32E-04	0.524966	0.532524	0.537243	0.54276	0.54667
3.93E-05	4.20E-04	0.542374	0.550879	0.554347	0.560112	0.563551
3.83E-05	8.18E-04	0.576276	0.584491	0.58862	0.591973	0.596829

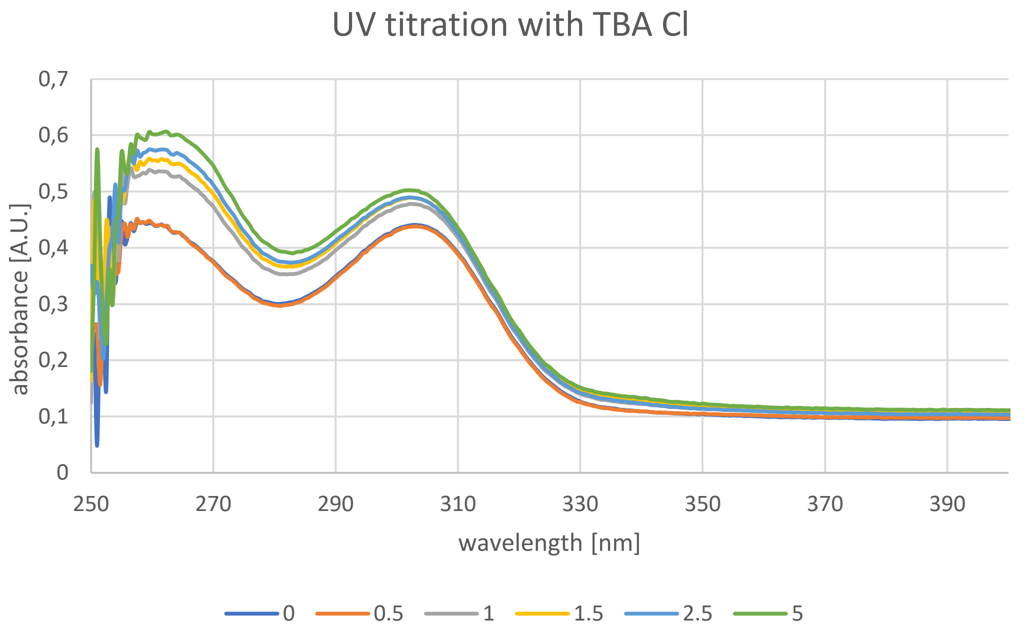

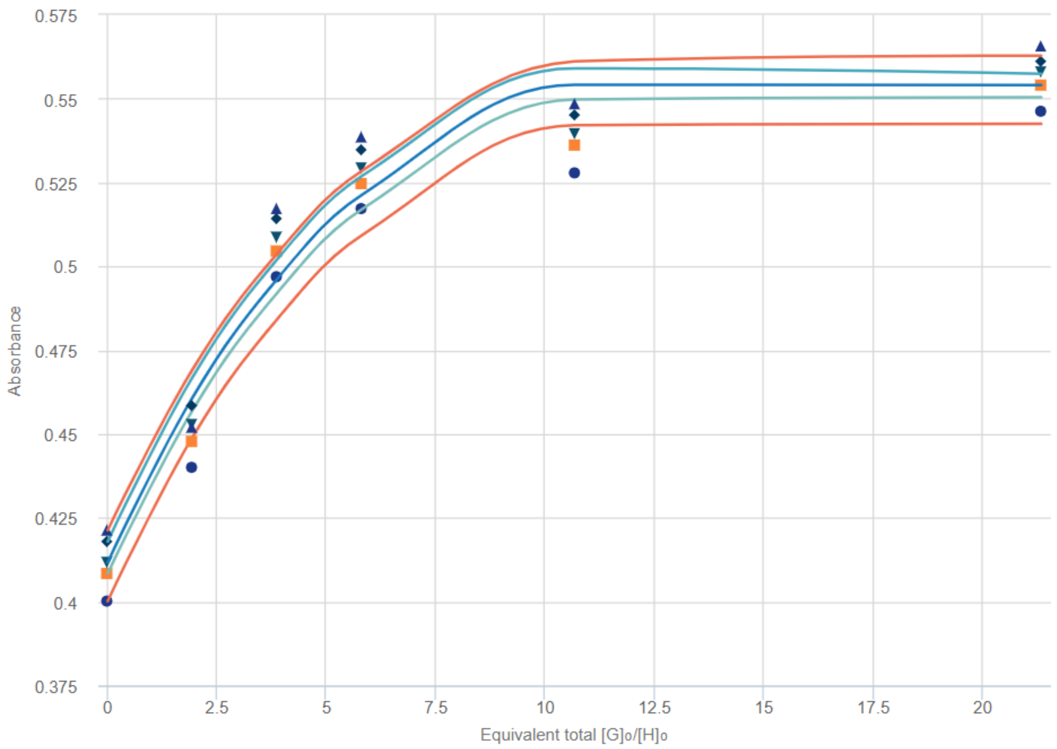

Figure S36. Compound **1c**, UV-Vis titration with TBA chloride, 25°C, DMSO, with depicted equivalents of aniont (top), .Bindfit calculated fit for selecte wavelengths (bottom).

c 1c [M]	c anion [M]	267 nm	267.5 nm	268 nm	268.5 nm	270 nm
4.04E-05	0.00E+00	0.418778	0.42552	0.430668	0.435897	0.440875
3.99E-05	1.84E-05	0.499088	0.507015	0.513801	0.519802	0.524964
3.94E-05	3.63E-05	0.538847	0.547417	0.554278	0.562341	0.568635
3.90E-05	5.38E-05	0.584561	0.595616	0.603534	0.611092	0.616868
3.85E-05	7.08E-05	0.610035	0.621444	0.629734	0.637534	0.643957
3.76E-05	1.04E-04	0.632645	0.643723	0.653215	0.660999	0.667758
3.68E-05	1.35E-04	0.647973	0.659025	0.668242	0.676455	0.682447
3.59E-05	1.65E-04	0.658271	0.669285	0.678515	0.686814	0.693302
3.52E-05	1.94E-04	0.679492	0.690956	0.70091	0.708379	0.714734
3.37E-05	2.48E-04	0.682428	0.694042	0.70344	0.71159	0.719153
3.23E-05	2.98E-04	0.683933	0.695112	0.704014	0.713589	0.720101
3.11E-05	3.43E-04	0.69809	0.709488	0.719584	0.728177	0.735325
2.99E-05	3.86E-04	0.700154	0.71239	0.722017	0.731151	0.738848
2.79E-05	4.62E-04	0.702706	0.715277	0.72467	0.734956	0.742626

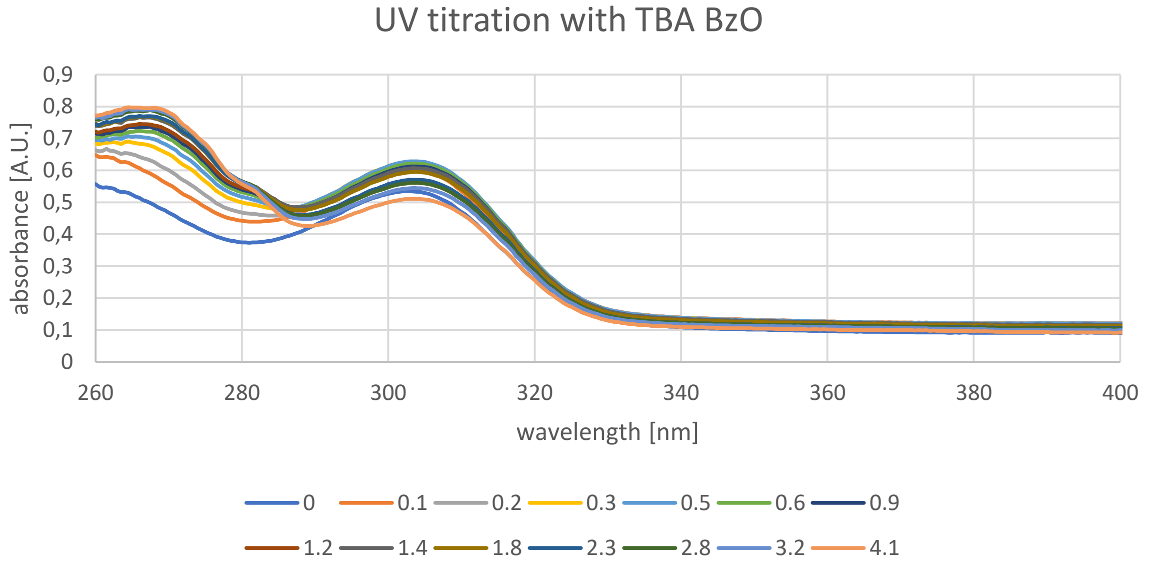

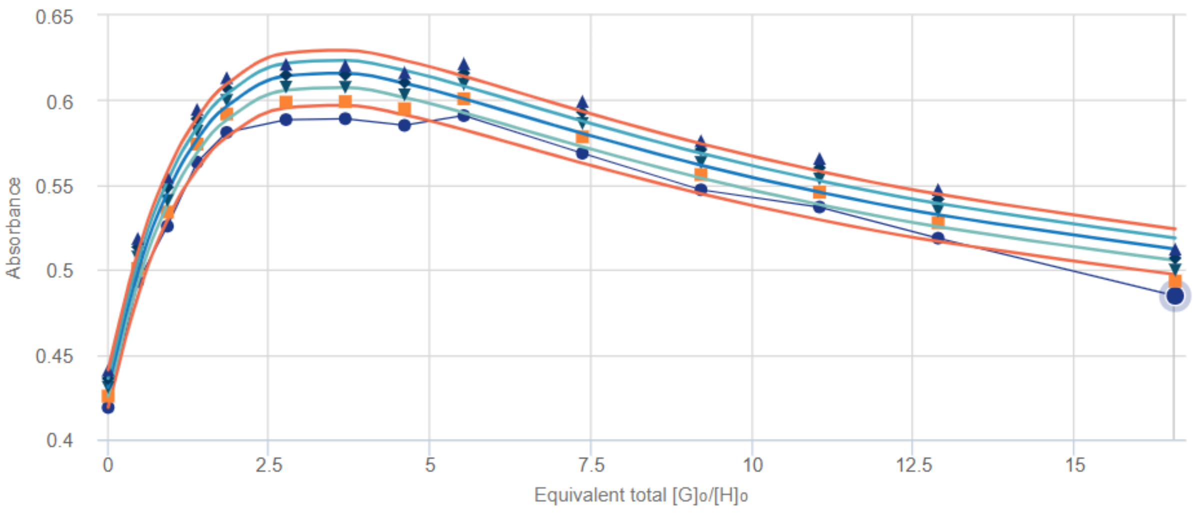

Figure S37. Compound **1c**, UV-Vis titration with TBA benzoate, 25°C, DMSO, with depicted equivalents of aniont (top), .Bindfit calculated fit for selecte wavelengths (bottom).

c 1c [M]	c anion [M]	267 nm	267.5 nm	268 nm	268.5 nm	270 nm
4.04E-05	0	0.427599	0.432048	0.438089	0.442649	0.447827
4.04E-05	7.37E-07	0.443989	0.448617	0.454966	0.45922	0.464014
4.04E-05	1.47E-06	0.444455	0.44963	0.455995	0.460462	0.465354
4.04E-05	2.21E-06	0.446822	0.452331	0.458563	0.463438	0.467609
4.02E-05	7.34E-06	0.459869	0.464907	0.469511	0.473419	0.476925
4E-05	1.46E-05	0.486251	0.489642	0.493483	0.497157	0.499542
3.98E-05	2.18E-05	0.504157	0.506765	0.5098	0.511458	0.513984
3.93E-05	3.95E-05	0.51735	0.519723	0.522185	0.523895	0.524938
3.89E-05	5.67E-05	0.526779	0.529467	0.531331	0.533306	0.534026
3.84E-05	7.36E-05	0.522772	0.524535	0.526411	0.527738	0.528818
3.8E-05	9E-05	0.522211	0.52392	0.526074	0.526589	0.527975
3.71E-05	0.000122	0.524877	0.526968	0.52897	0.529713	0.530305
3.63E-05	0.000152	0.516381	0.518749	0.519371	0.520805	0.521296
3.47E-05	0.000209	0.510186	0.511717	0.513282	0.514768	0.515538
3.33E-05	0.000261	0.501151	0.503164	0.505108	0.50569	0.506375

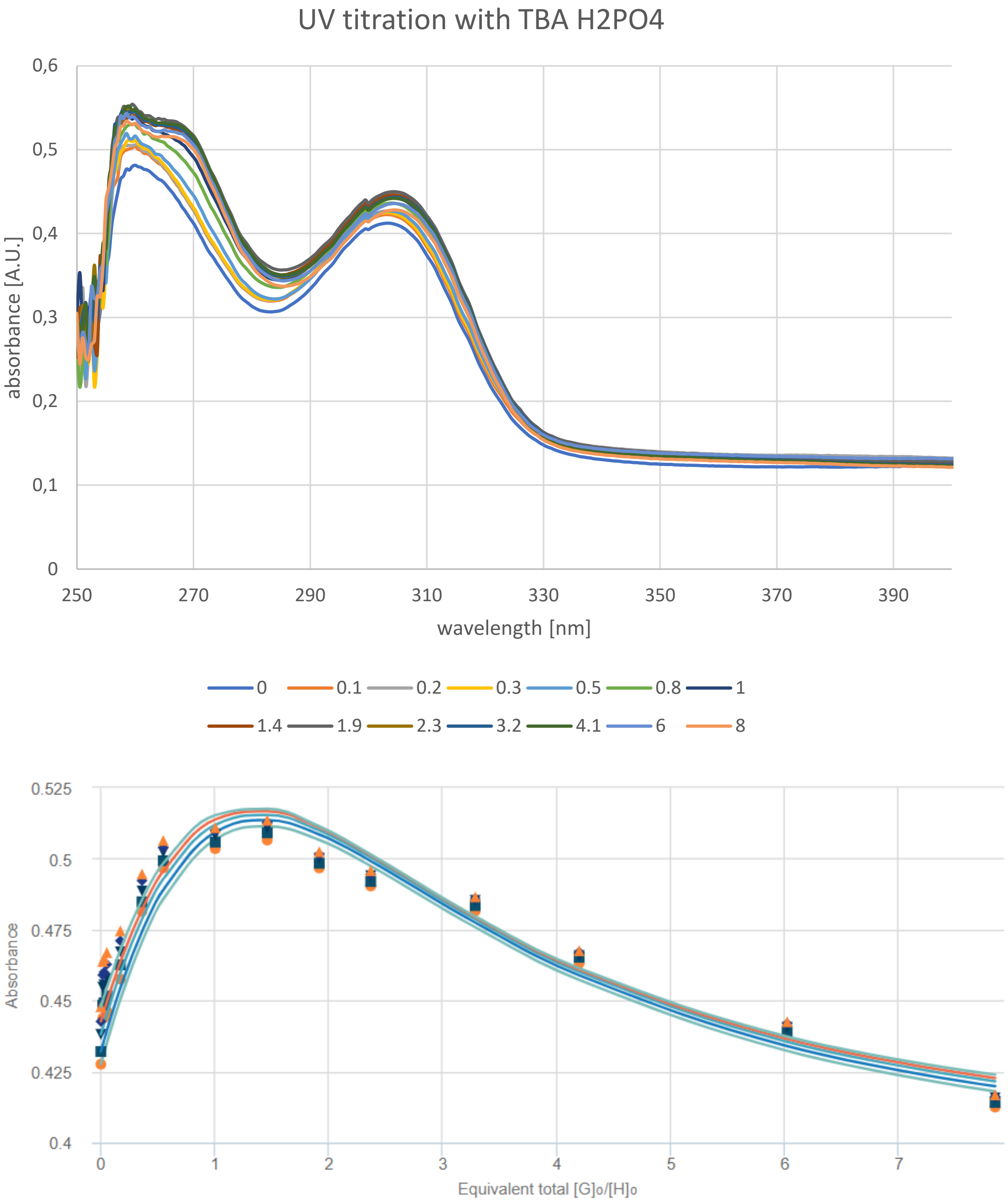

Figure S38. Compound **1c**, UV-Vis titration with TBA dihydrogen phosphate, 25°C, DMSO, with depicted equivalents of aniont (top), .Bindfit calculated fit for selecte wavelengths (bottom).

M = 278.35 g.mol^{-1}, monoclinic system, space group *P*2$_1$/*c*, *a* = 20.6893 (6) Å, *b* = 8.8817 (2) Å, *c* = 8.2553 (2) Å, *β* = 97.8399 (11)°, *Z* = 4, *V* = 1502.24 (7) Å^3, D_c = 1.231 g.cm^{-3}, *μ*(Cu-Kα) = 0.70 mm^{-1}, crystal dimensions of 0.56 × 0.28 × 0.25 mm. Data were collected at 250 (2) K on a Bruker D8 Venture Photon CMOS diffractometer with Incoatec microfocus sealed tube Cu-*Kα* radiation. The structure was

solved by charge flipping methods[1] and anisotropically refined by full matrix least squares on F squared using the CRYSTALS[2] to final value $R = 0.039$ and $wR = 0.115$ using 2848 independent reflections (ϑ_{max} = 70.3°), 181 parameters and 0 restrains. The hydrogen atoms bonded to carbon atoms were placed in calculated positions and refined with a riding constrains. MCE[3] was used for visualization of electron density maps. Deposition Number CCDC 2385194 contain the supplementary crystallographic data for this paper. These data are provided free of charge by the joint Cambridge Crystallographic Data Centre and Fachinformationszentrum Karlsruhe http://www.ccdc.cam.ac.uk/structures.

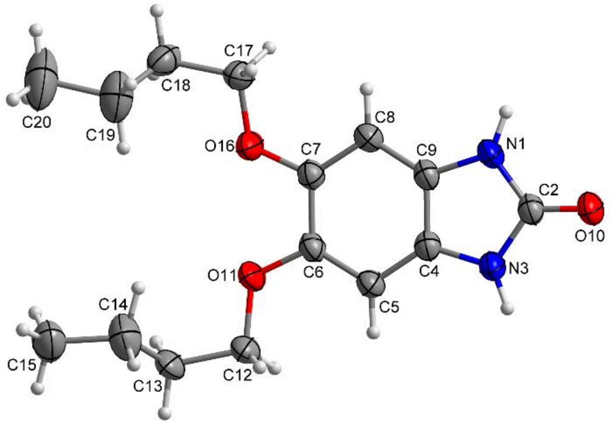

Figure S39. Crystallographic data for compound ***7***, the ADPs drawn at 50% probability level.

[1] Palatinus, L., Chapuis, G. (2007). J. Appl. Cryst. 40, 786-790. DOI: 10.1107/S0021889807029238

[2] Betteridge, P.W., Carruthers, J.R., Cooper, R.I., Prout, K. & Watkin, D.J. (2003). J. Appl. Cryst. 36, 1487. DOI: 10.1107/S0021889803021800

[3] Rohlicek J., Husak M. (2007) J. Appl. Cryst. 40, 600-601 DOI: 10.1107/S0021889807018894
